# Connections of the juxtaventromedial region of the lateral hypothalamic area in the male rat

**DOI:** 10.3389/fnsys.2015.00066

**Published:** 2015-05-27

**Authors:** Joel D. Hahn, Larry W. Swanson

**Affiliations:** Department of Biological Sciences, University of Southern CaliforniaLos Angeles, CA, USA

**Keywords:** lateral hypothalamic area, fundamental behavior, cognitive systems, motor systems, hypothalamus

## Abstract

Evolutionary conservation of the hypothalamus attests to its critical role in the control of fundamental behaviors. However, our knowledge of hypothalamic connections is incomplete, particularly for the lateral hypothalamic area (LHA). Here we present the results of neuronal pathway-tracing experiments to investigate connections of the LHA juxtaventromedial region, which is parceled into dorsal (LHAjvd) and ventral (LHAjvv) zones. *Phaseolus vulgaris* leucoagglutinin (PHAL, for outputs) and cholera toxin B subunit (CTB, for inputs) coinjections were targeted stereotaxically to the LHAjvd/v.

**Results**: LHAjvd/v connections overlapped highly but not uniformly. Major joint outputs included: Bed nuc. stria terminalis (BST), interfascicular nuc. (BSTif) and BST anteromedial area, rostral lateral septal (LSr)- and ventromedial hypothalamic (VMH) nuc., and periaqueductal gray. Prominent joint LHAjvd/v input sources included: BSTif, BST principal nuc., LSr, VMH, anterior hypothalamic-, ventral premammillary-, and medial amygdalar nuc., and hippocampal formation (HPF) field CA1. However, LHAjvd HPF retrograde labeling was markedly more abundant than from the LHAjvv; in the LSr this was reversed. Furthermore, robust LHAjvv (but not LHAjvd) targets included posterior- and basomedial amygdalar nuc., whereas the midbrain reticular nuc. received a dense input from the LHAjvd alone. Our analyses indicate the existence of about 500 LHAjvd and LHAjvv connections with about 200 distinct regions of the cerebral cortex, cerebral nuclei, and cerebrospinal trunk. Several highly LHAjvd/v-connected regions have a prominent role in reproductive behavior. These findings contrast with those from our previous pathway-tracing studies of other LHA medial and perifornical tier regions, with different connectional behavioral relations. The emerging picture is of a highly differentiated LHA with extensive and far-reaching connections that point to a role as a central coordinator of behavioral control.

## Introduction

About a century of research on the hypothalamic neuronal network has established its critical role in the control of fundamental behaviors and their supporting homeostatic processes (for historical reviews see Le Gros Clark, 1938; Fulton et al., 1940; Harris, 1955; Nauta and Haymaker, 1969). The early research established a broad functional role for the hypothalamus, and indicated further that different hypothalamic regions could control different functions (or different aspects of the same function). However, despite the profound behavioral and physiological effects of experimental hypothalamic electrolytic lesion and electrical stimulation, nothing definitive was said about the organization of the underlying neuronal connections—although much was hinted at in pioneering neuroanatomical studies using a variety of now classic histological staining techniques (Gurdjian, 1927; Krieg, 1932; Ramon y Cajal, 1995). The major limitation on the acquisition of connection data was an absence of techniques for determining neuronal connections (such as anterograde and retrograde pathway-tracing) and neuronal chemoarchitecture (such as immunohistochemistry—IHC). Prior to the emergence and (beginning in the 1970s) application of these methods to neuroanatomical studies, the available and prevailing techniques were axon degeneration methods and a variety of histological staining techniques (for further perspective and reviews see Haymaker et al., 1969; Morgane and Panksepp, 1979; Swanson, 1987, 1999; Zaborszky et al., 2006).

Initial non-lesion anterograde neuronal pathway-tracing studies employed the autoradiographic method (tritiated amino acids) which, despite problems with interpretability (Swanson, 1981), was applied extensively to investigate central connections, including those of the LHA (Saper et al., 1979; Veening et al., 1982). In parallel with advances in pathway-tracing methods, advances in neuropharmacology led to the availability of receptor-targeted drugs which could replace electrical stimulation methods to identify central sites of functional significance—for example, the determination of central sites from which drinking could be elicited by central injections of either angiotensin II, or the cholinergic agonist carbachol (Swanson and Sharpe, 1973; Swanson et al., 1973; Sharpe and Swanson, 1974).

As neuropharmacological methods enabled more selective targeting than electrical stimulation, so the introduction of non-isotopic retrograde and anterograde neuronal pathway-tracers provided more effective tools than lesion/degeneration methods with which to determine the organization of central neuronal connections. Prominent among the latter is the lectin *Phaseolus vulgaris* leucoagglutinin (PHAL), which was introduced as an anterograde neuronal tracer in the 1980s (Gerfen and Sawchenko, 1984), and enabled for the first time (with detection by IHC) the qualitative determination of the microscopic morphology and topographic organization of connections between stereotaxically targeted gray matter regions. Similarly, now routinely used retrograde neuronal tracers introduced in the same period include the B subunit of cholera toxin (CTB) (Stoeckel et al., 1977; Dumas et al., 1979; Trojanowski et al., 1981; Luppi et al., 1987, 1990), and hydroxystilbamidine (trade name Fluoro-Gold; FG) (Schmued and Fallon, 1986).

In the past decade, a second revolution in methods for system-level connection analysis has occurred, comparable in impact to that which occurred in the 1970s (Swanson, 2007). The application of molecular genetics methods (such as opto- and pharmacogenetic, and viral pathway-tracing approaches) to investigating hypothalamic structure-function relations is beginning to provide long-sought clarity about the organization of this critical neuronal network. Recent investigations that exemplify the application of these methods include the elucidation of hypothalamic networks for feeding behavior (Betley et al., 2013), and aggression (Lin et al., 2011)—for reviews of the approaches see (Zaborszky et al., 2006; Fenno et al., 2011; Farrell and Roth, 2013; Sternson, 2013). Nevertheless, now classic pathway-tracing techniques, using tracers such as PHAL and CTB, remain useful tools in the continuing quest to determine the basic plan of the hypothalamic neuronal network, and by extension the nervous system (Swanson, 2000, 2007; Zingg et al., 2014). Furthermore, analysis and interpretation of the massive amount of connectional data generated by the application of these techniques is increasingly being aided by computational neuroinformatics approaches (Swanson and Bota, 2010; Brown and Swanson, 2013; Bota et al., 2014).

In two previous studies on the connections of the LHA, we employed a CTB + PHAL co-injection strategy to investigate the macroconnections of LHA regions medially and dorsally adjacent to the column of the fornix: The LHA juxtadorsomedial and juxtaparaventricular regions (LHAjd, and LHAjp), and the LHA suprafornical region (LHAs). Impetus for these studies was generated by a novel provisional cytoarchitectural parcellation schema for the LHA (Swanson, 2004), and they served as starting point for more focused structure-functional analysis of individual components. An earlier PHAL study focused on an LHA region ventral to the fornix-LHA subfornical (LHAsf) (Goto et al., 2005).

A notable finding was the sheer number of gray matter regions connecting to the delineated LHA regions (up to several hundred, far exceeding the first-order connectivity of any other brain region studied similarly to date). In addition, distinct topographic differences were found. Relating the prominent connections of the LHAsf anterior part (LHAsfa), LHAjp, LHAjd, and LHAs to the existing literature, possible behavioral functions were suggested to which these connections may contribute. In very broad terms these hypothesized functions were primarily ingestive behavior (for the LHAs), defensive behavior for LHAjp and LHAjd (Hahn and Swanson, 2010, 2012), and defensive or foraging behavior for the LHAsf (Goto et al., 2005). Two recent functional studies have provided data consistent with these hypotheses: LHAjd neurons are indicated to play a role in the expression of conditioned defensive responses (Faturi et al., 2014), and LHAs neurons in the activation of feeding in response to activation of agouti-related peptide (AGRP)-expressing axons in the LHAs (Betley et al., 2013).

In the present study we focus on the LHA region ventromedial to the fornix (and adjacent to the hypothalamic ventromedial nucleus—VMH), that is divided into two zones: The LHA juxtaventromedial region, dorsal- (LHAjvd) and ventral (LHAjvd) zones. The parcellation of these dorsoventrally contiguous zones, based on the Nissl cytoarchitecture, was described previously (Swanson, 2004). Briefly, their rostral to caudal extent is approximately the same as the VMH (which also provides a medial boundary), and they extend laterally to the LHAsf. The LHAjvd is bounded dorsally by the LHAjd and LHAjp; the LHAjvv is bounded ventrally by the tuberal nucleus. In addition to their cytoarchitectural parcellation, the tract of the post-commissural fornix provides a readily identifiable fiducial marker that corresponds approximately to the dorsolateral boundary of the LHAjvd.

## Methods

The methods follow those described previously (Hahn and Swanson, 2010, 2012), and are provided here abridged. Experiments were performed according to the NIH Guidelines for the Care and Use of Laboratory Animals, and all protocols were approved by the University of Southern California Institutional Animal Care and Use Committee. Adult male Sprague–Dawley rats (290–360 g; Harlan) under anesthesia (1 ml/kg body weight of 50 mg/ml ketamine and 10 mg/ml xylazine, intramuscular) received single, iontophoretic injections of a mixture of 2.5% *P. vulgaris* leucoagglutinin (PHAL; Vector Labs) and 0.25% cholera toxin B subunit (CTB; List Labs) targeted stereotaxically to the LHAjv region (Swanson, 2004). From 12 to 20 days later, the rats were deeply anesthetized with sodium pentobarbital (40 mg/kg body weight, intraperitoneal) and perfused with ice-cold 0.9% saline followed by 4% paraformaldehyde (pH 9.5). Brains were removed and post-fixed overnight at 4°C in the same fixative containing 12% sucrose, and then frozen rapidly in dry-ice cooled hexane. Serial 25 or 30 μm thick transverse-plane frozen sections (4 or 5 series) were obtained with the use of a sliding microtome. One series was processed for immunohistochemical (IHC) detection of PHAL, another for detection of CTB, and an intervening series was stained with cresyl violet (Nissl stain) to reveal cytoarchitecture (Simmons and Swanson, 1993). For IHC detection of tracers, the sections were incubated in primary antibodies directed against either CTB (1:10,000; goat, List BioLabs) or PHAL (1:3000; rabbit, Dako) (3 nights, refrigerated). This was followed by a biotinylated secondary antibody (1:1000; donkey anti-rabbit or goat, Vector Labs) (90 min) and then an avidin-biotin-horseradish peroxidase reagent (1:1000; ABC reagent, Vector Labs) (2 h). The sections were then recycled to the secondary antibody (overnight, refrigerated), and the following day placed in freshly prepared ABC reagent (90 min). Visualization of labeling was accomplished with the use of 3,3′-Diaminobenzidine (DAB, 0.05%) in the presence of hydrogen peroxide (0.005%), with the addition of ammonium nickel (II) sulfate (0.1%) to enhance visualization of CTB. Injection sites (PHAL–containing perikarya, and CTB deposits) were identified along with anterogradely (PHAL) labeled axons, and retrogradely (CTB) labeled perikarya; these were analyzed and plotted (Illustrator CS5/6, Adobe) onto a digital reference series of drawings of the rat brain as described previously (Swanson, 2004; Hahn and Swanson, 2010). Adjacent series of Nissl–stained sections were used for reference. Digital photomicrographs [Microscope: Zeiss AxioImager (Carl Zeiss); Cameras: Orca ER (Hamamatsu), or Retiga 2000R (Q Imaging)] were acquired singly or using stitching software (Volocity, Perkin Elmer) and then composed (Photoshop CS5/6, Adobe).

## Results

**Figure 1:**
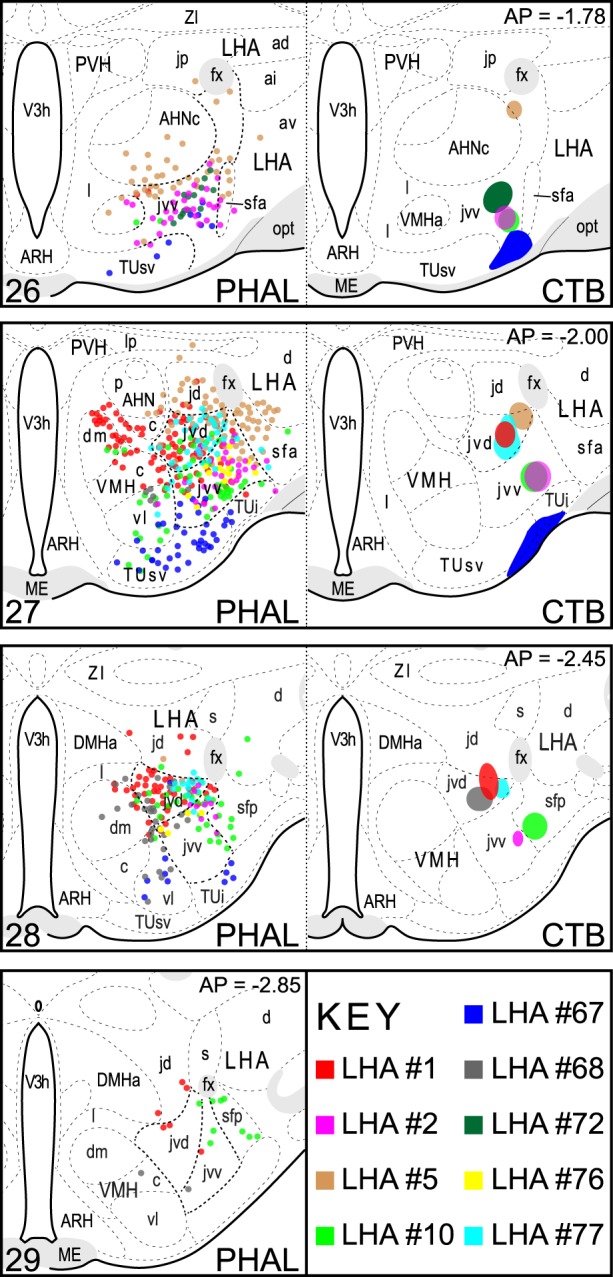
**LHAjv region injection site maps**. The extent of injection sites for nine CTB + PHAL coinjections are represented. These included the LHAjvd or LHAjvd, and neighboring regions. Data were plotted with the aid of a *drawing tube* and with reference to adjacent Nissl-stained sections. Numbers in the upper right/lower left corresponding (respectively) to distance caudal Bregma/atlas levels (Swanson, 2004). This figure is also available as a separate vector graphics file (Figure S1).

**Figure 2:**
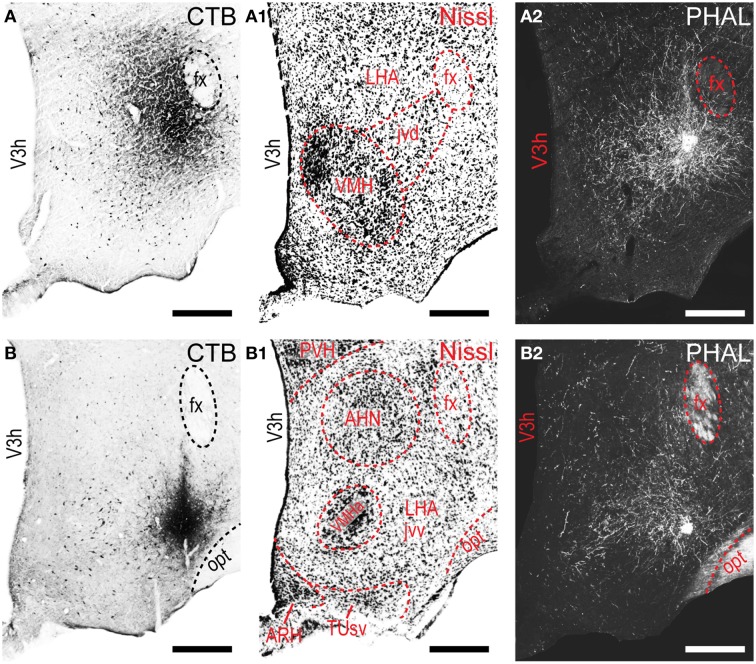
**LHAjvd and LHAjvv representative injection sites**. Immunohistochemically detected CTB (brightfield) and PHAL (darkfield) coinjection sites centered in the LHAjvd **(A,A2)** and LHAjvv **(B,B2)** at the levels shown. Adjacent Nissl-stained sections are also shown (LHAjvd: **A1**; LHAjvv: **B1**). NB. Apparent further extent of CTB injection site shown in “**A**” compared to “**B**” is an artifact resulting from partial dispersion of nickel-enhanced DAB reaction product during the time between reaction and section mounting, attributable to weaker fixation—the darker center area of the injection site in “**A**” is representative of its essential extent at this level (see Figure 1). Scale bars = 250 μM.

From a series of PHAL + CTB coinjection experiments targeted to the LHA (*n* = 93), the spatial extent of nine injection sites resulting from coinjections that included the LHAjvv and/or LHAjvd (LHAjvd/v) is shown in Figure 1. The most comprehensive analysis was performed on two representative datasets, resulting from experiments with injection sites that were the most restricted to (and inclusive of) the LHAjvv (experiment LHA #2) or the LHAjvd (experiment LHA #77) (Figures 1, 2).

**Figure 3:**
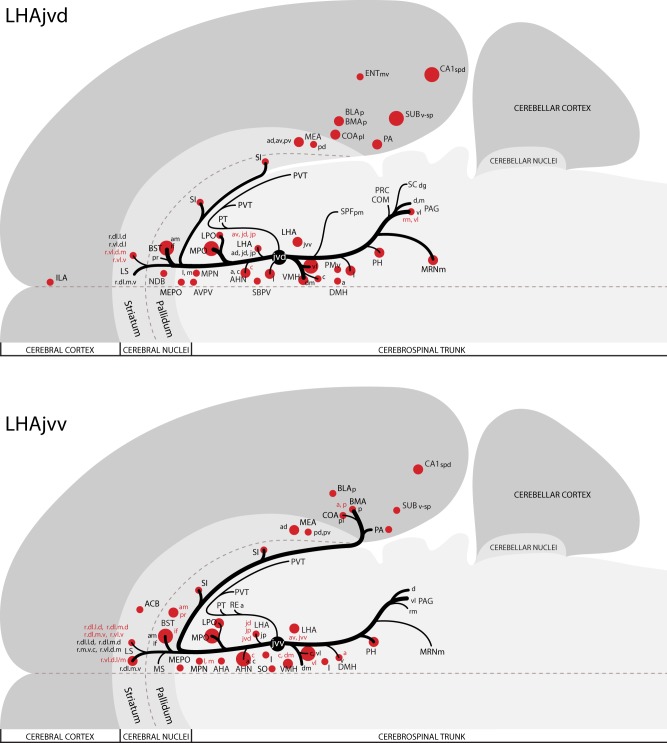
**Summary of major LHAjv region connections**. General organization of representative principal connections (moderate or higher in magnitude) of the LHAjvd (experiment LHA #2) and LHAjvv (experiment LHA #77) plotted onto a truncated flatmap representation of the central nervous system (see Swanson, 2004). Axonal outputs are represented by black lines; sites of retrograde labeling (inputs) by red discs. The relative magnitude of each connection is indicated by line thickness/disc diameter. Red text is used to indicate sites of LHAjvd/v input for instances where different subdivisions of the same region have different input and output connections. This figure is also available as a separate vector graphics file (Figure S3).

**Figure 4:**
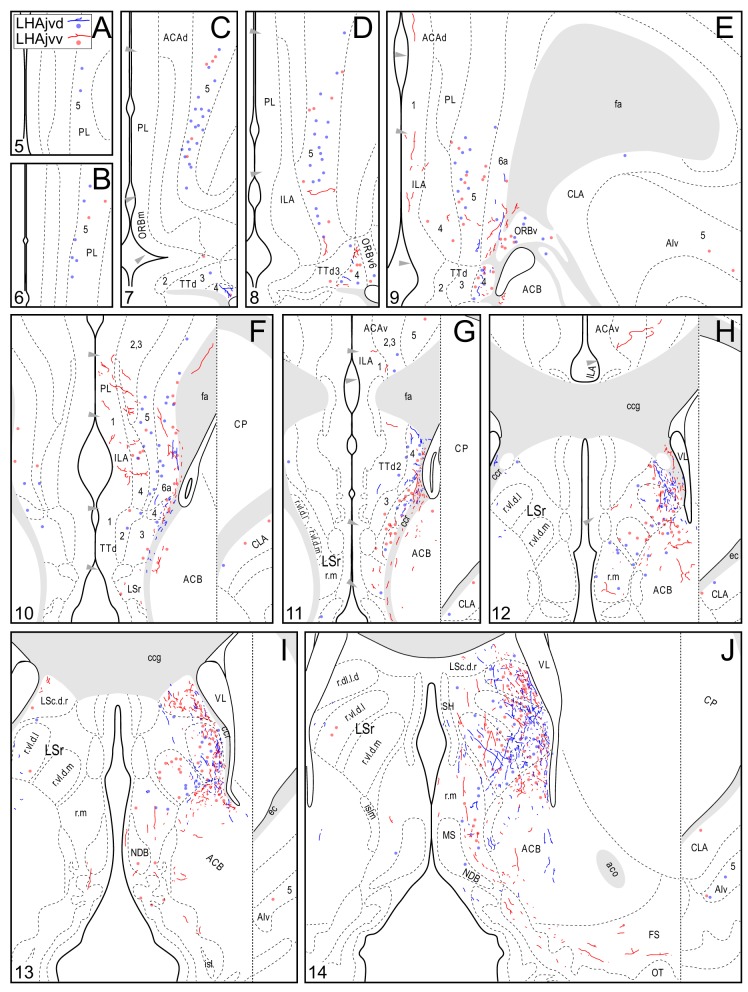

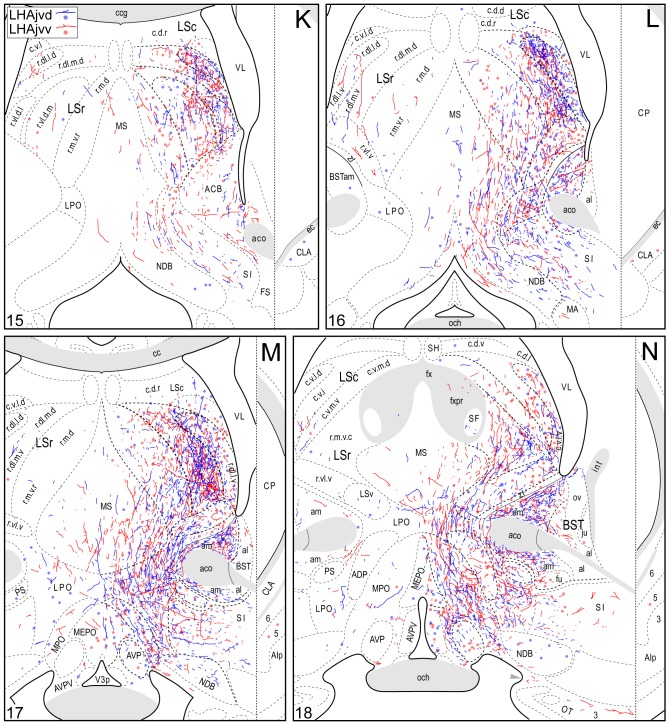

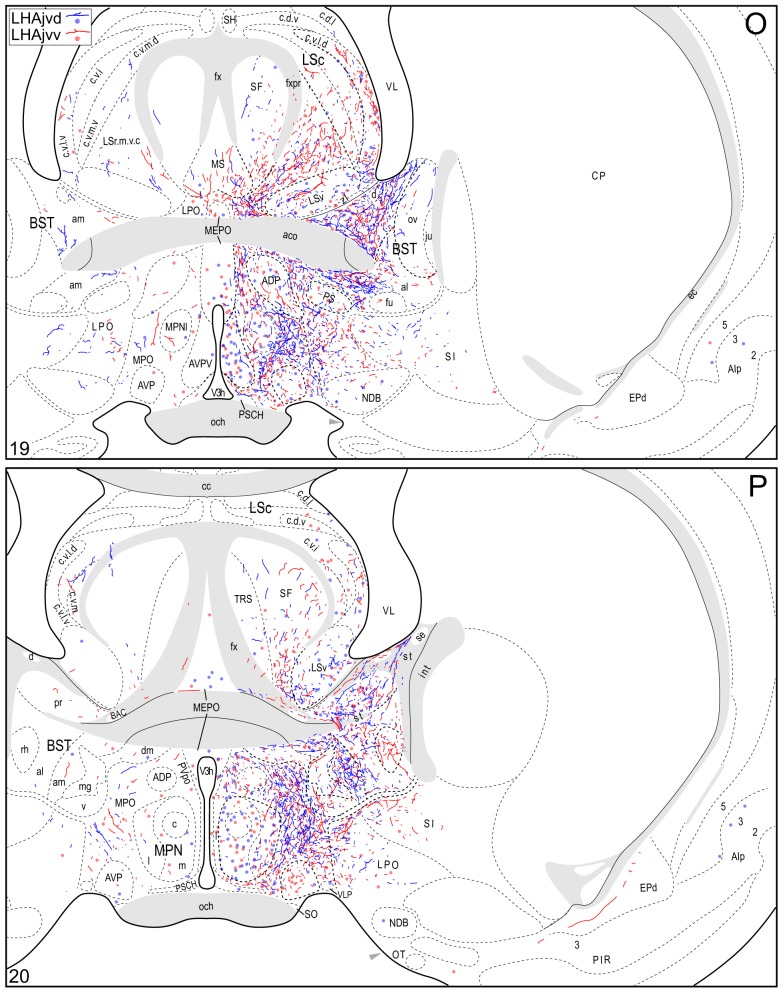

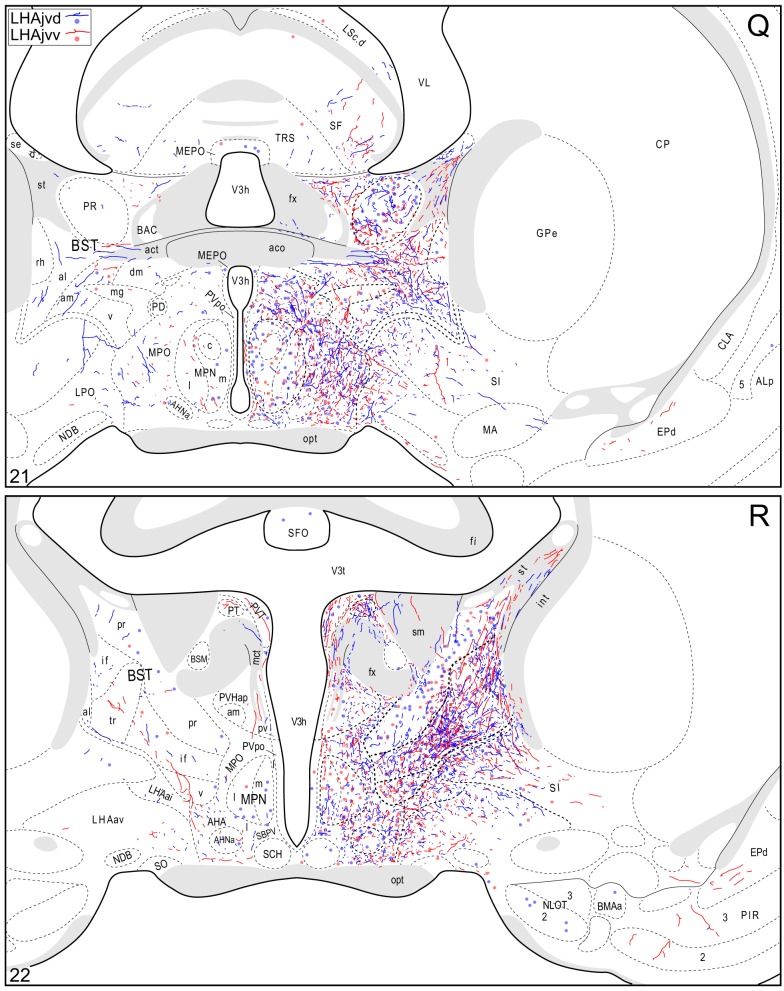

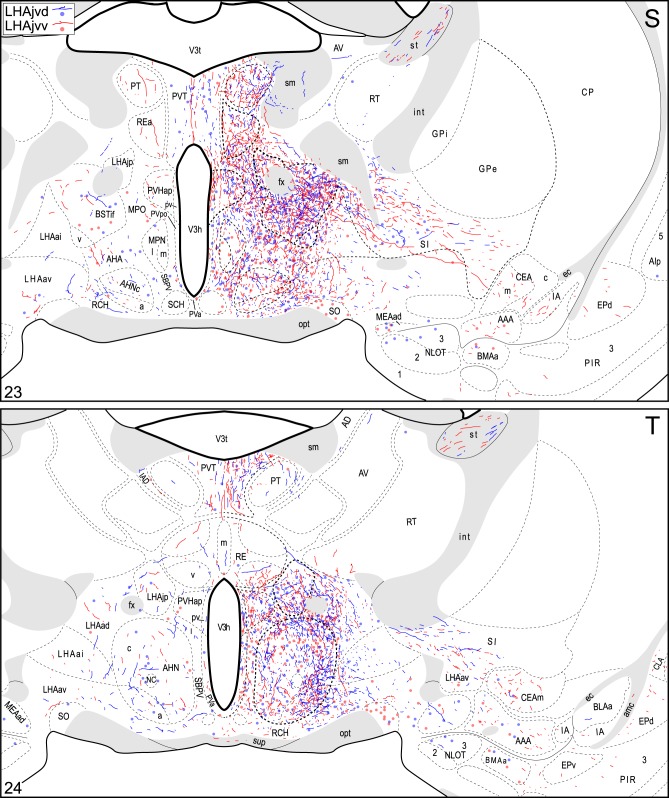

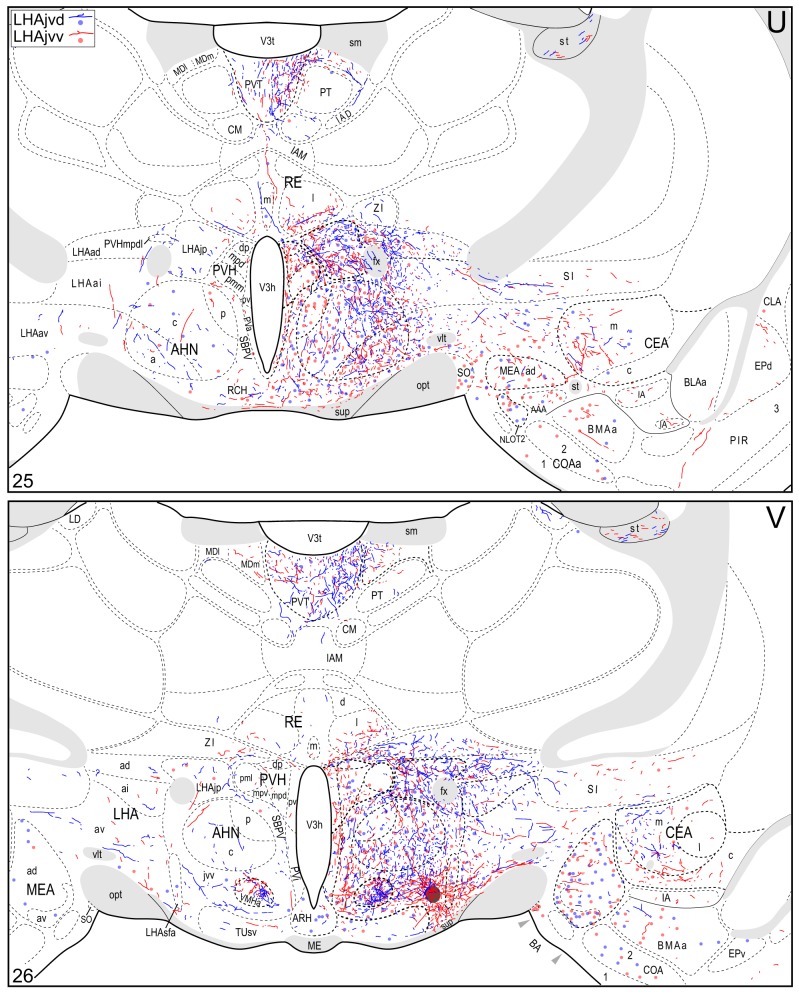

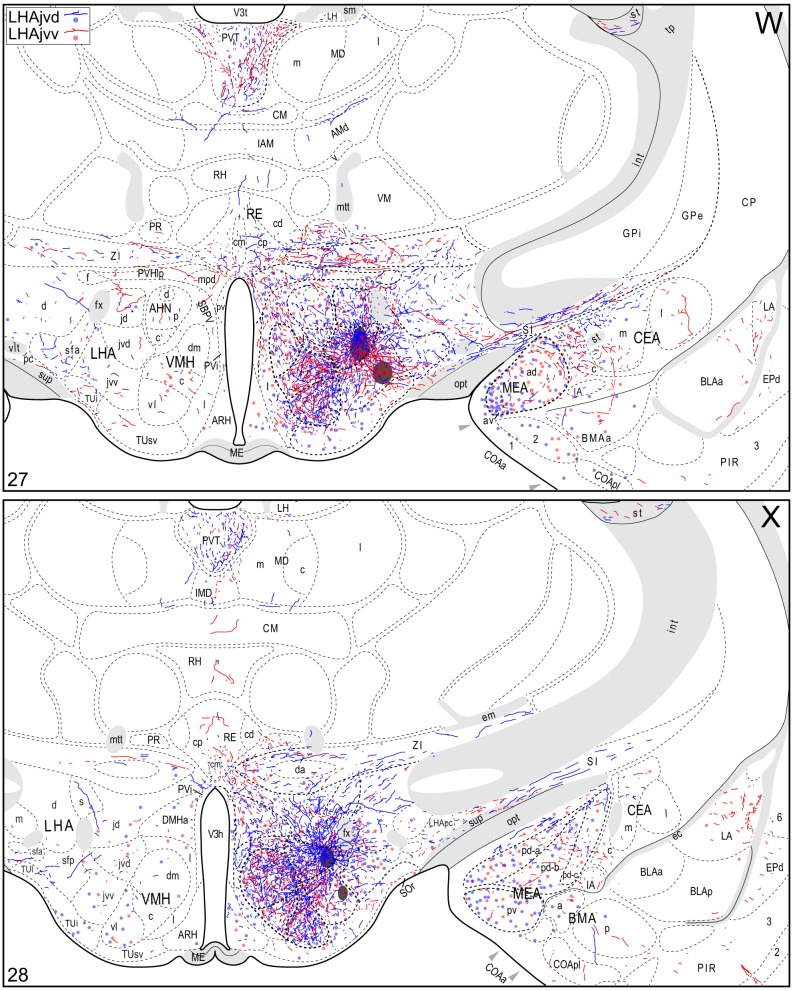

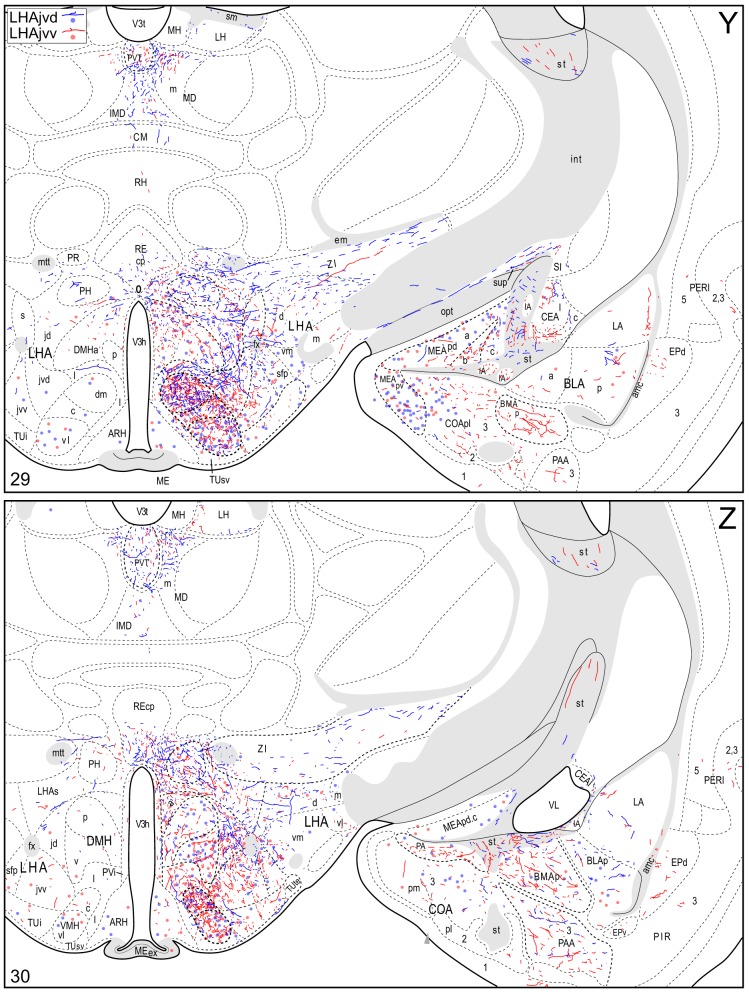

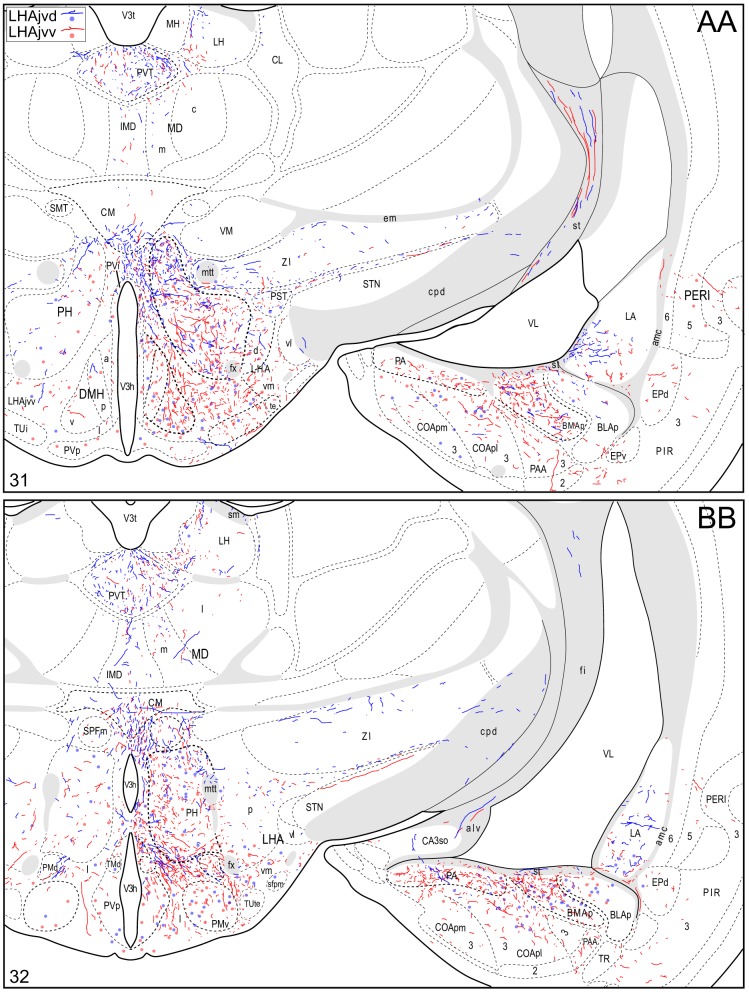

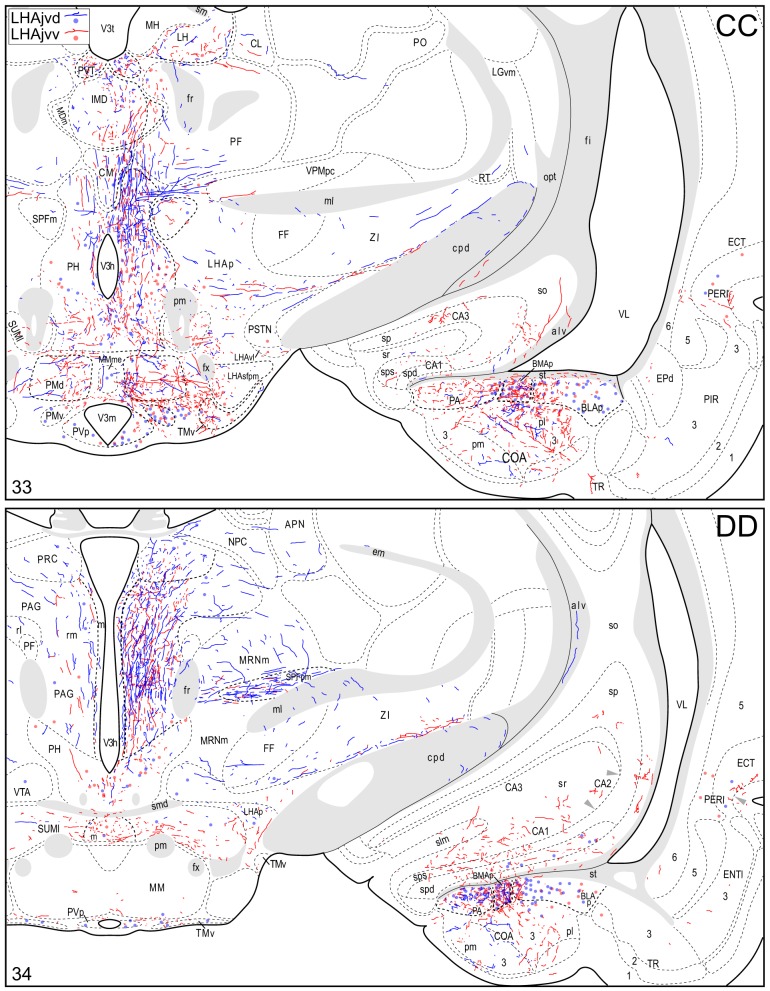

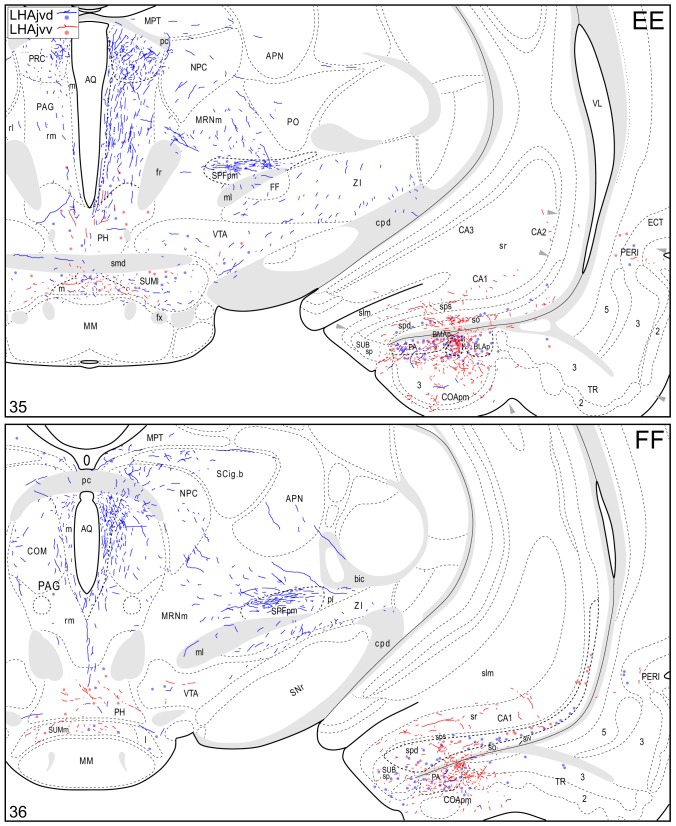

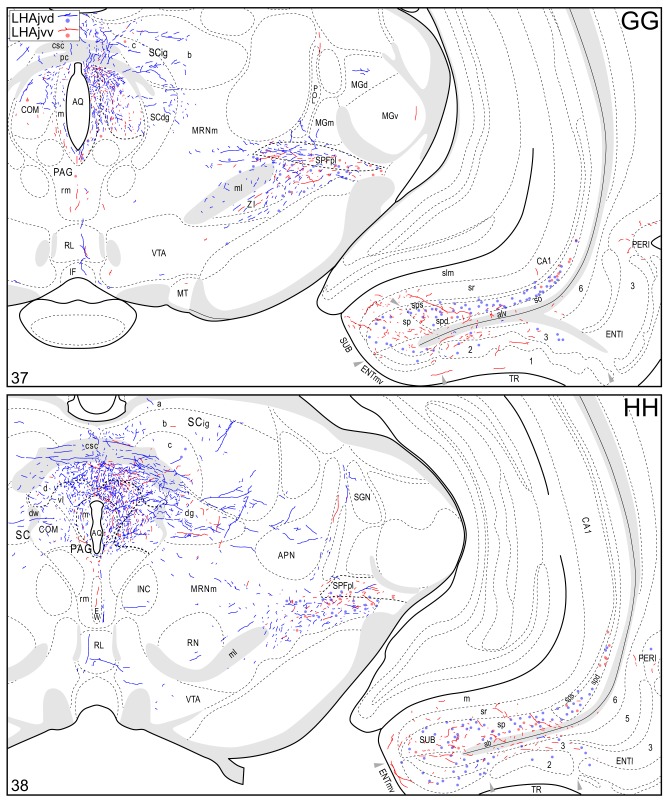

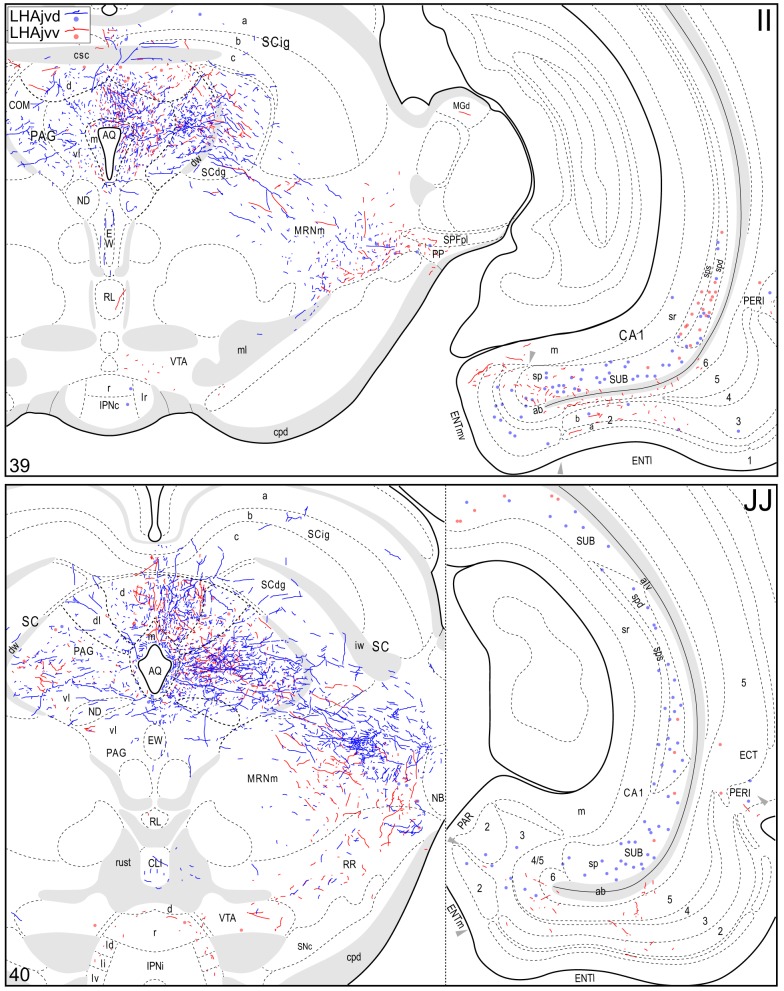

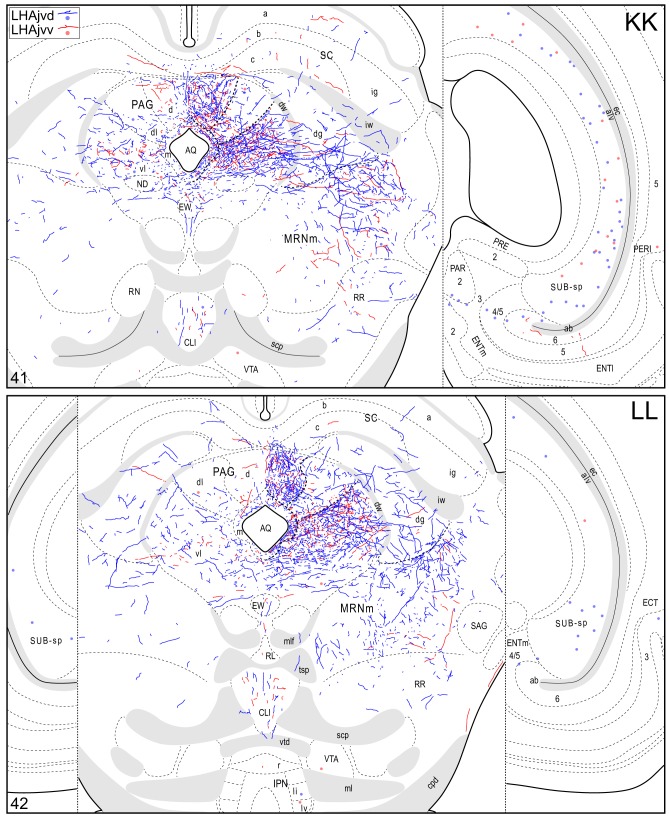

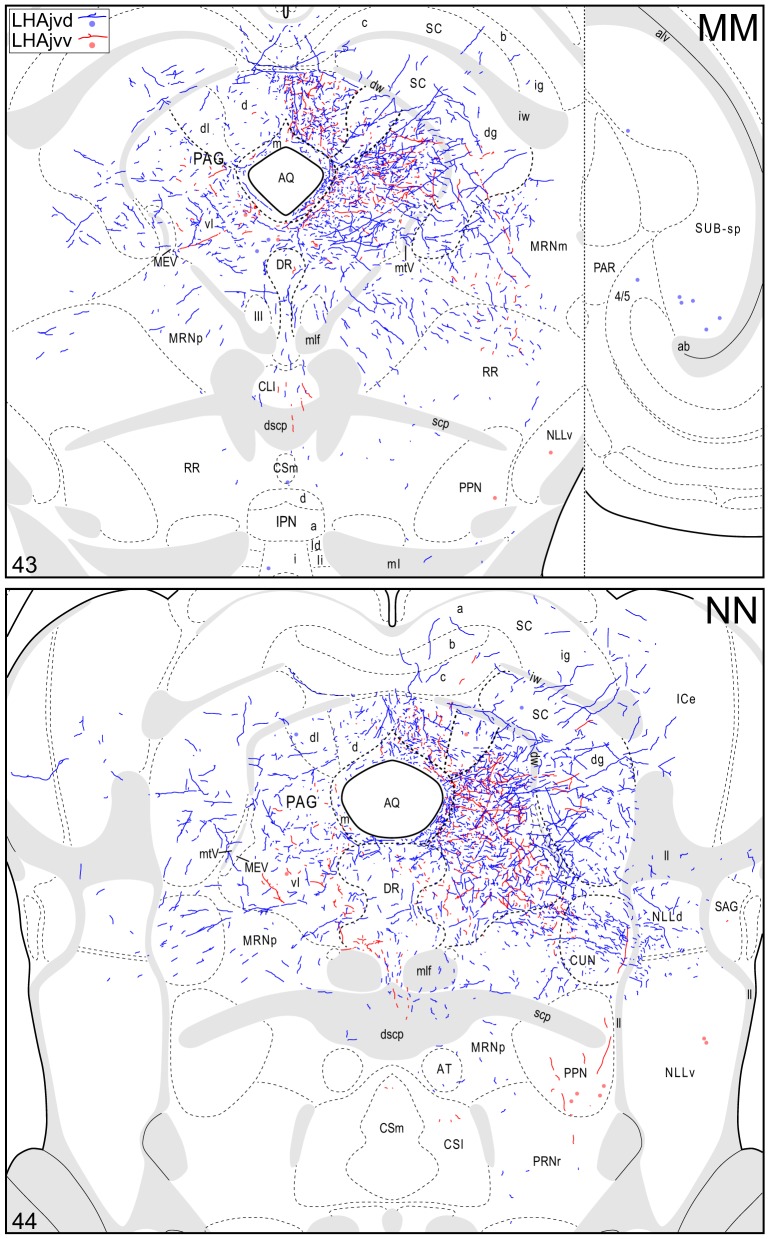

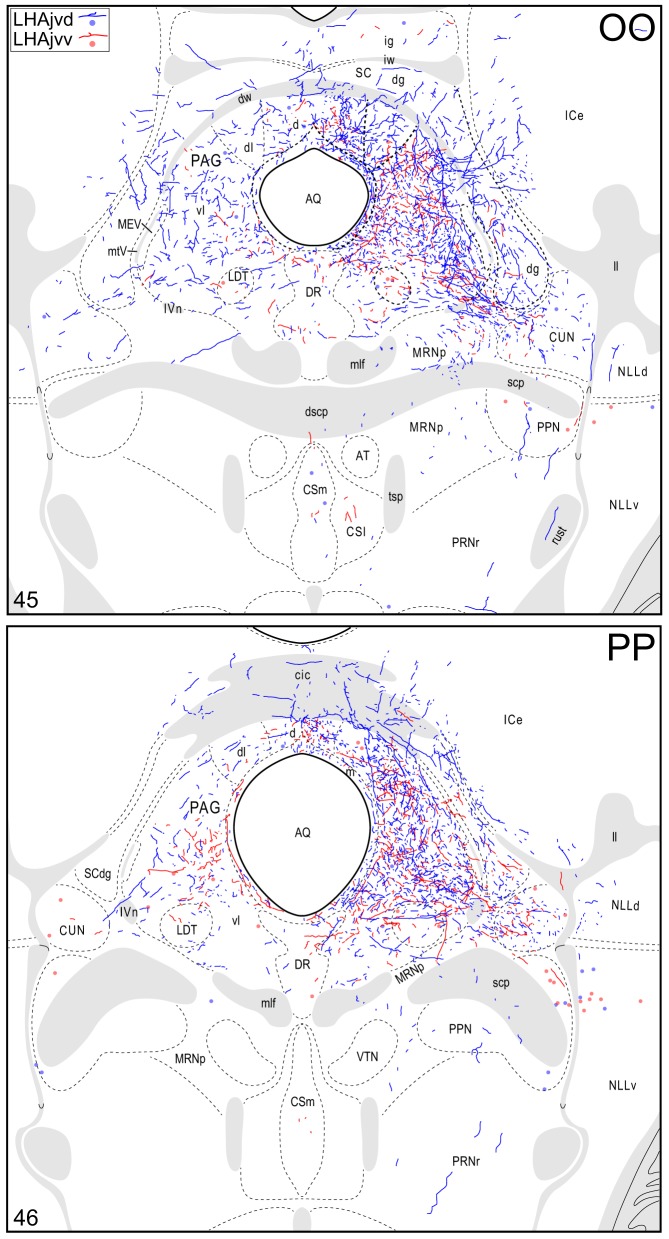

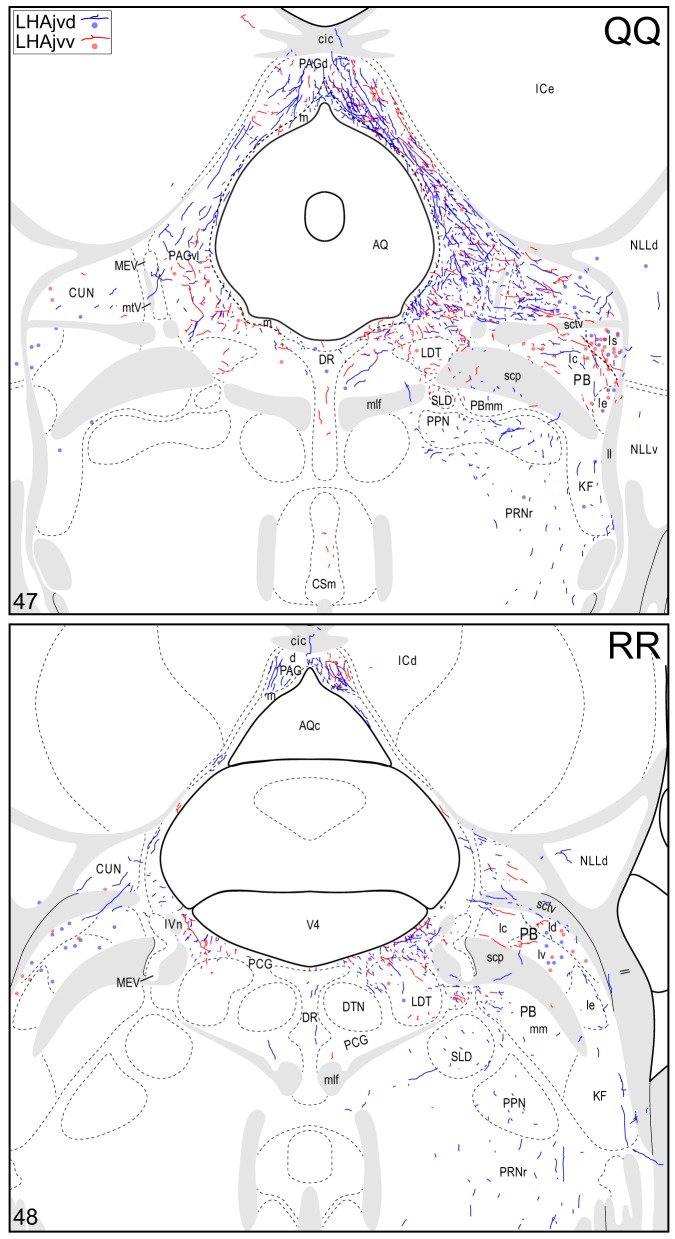

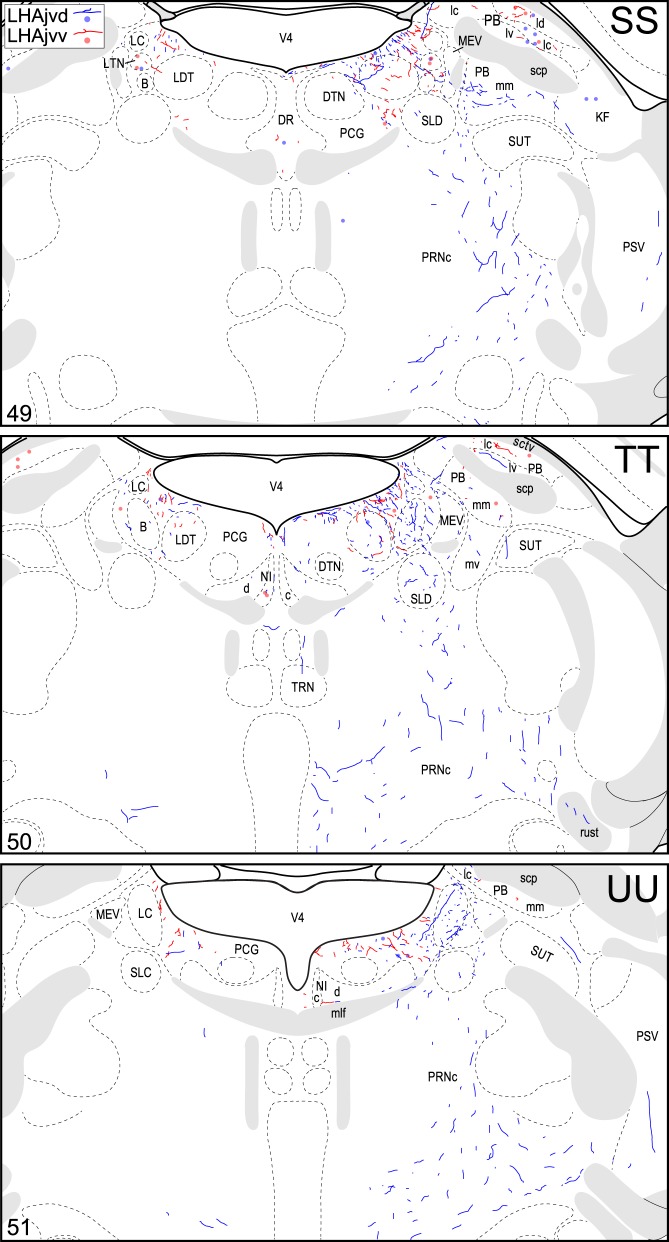

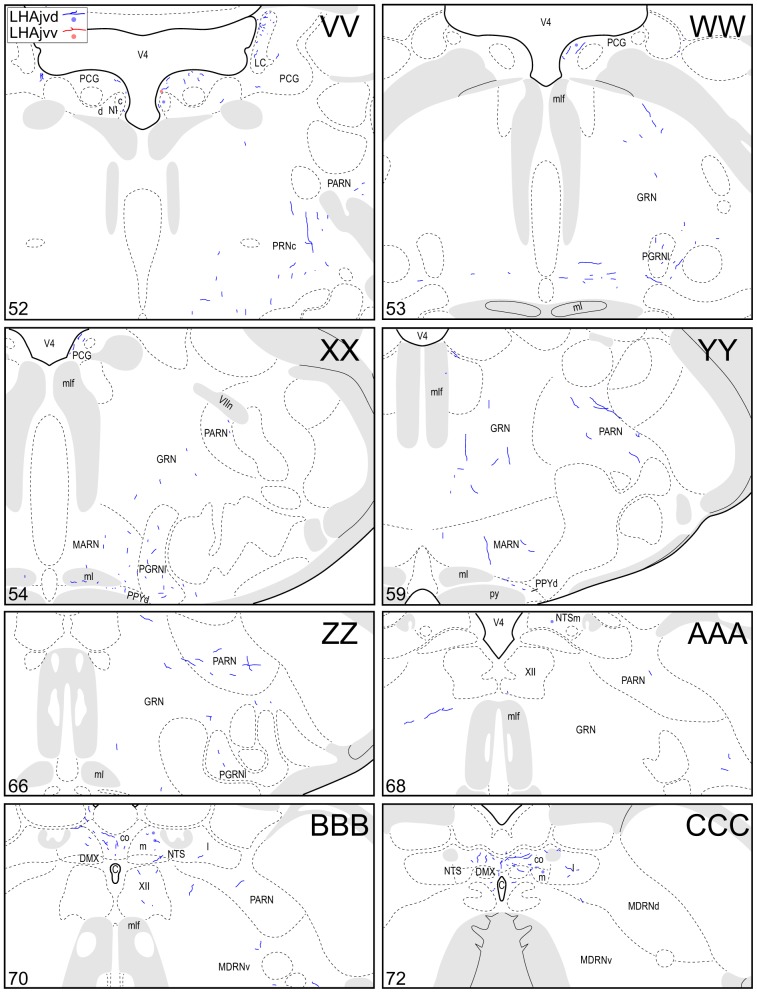
**Connection maps of the LHAjvd and LHAjvv**. Representative neuronal connections (inputs and outputs) of the LHAjvd (experiment LHA #2) and LHAjvv (experiment LHA #77) mapped to reference atlas levels (Swanson, 2004). PHAL labeled axons and CTB labeled cell bodies were visualized immunohistochemically and mapped with reference to adjacent Nissl-stained sections. Colored lines represent axonal output connections (PHAL); colored dots represent individual retrogradely labeled cell bodies (CTB); blue = LHAjvd, red = LHAjvv. Dots are semi-transparent to facilitate visualization of superimposed cell bodies. NB. During sectioning of brain tissue blocks from experiment LHA #77, a small portion of tissue was lost at the block faces, corresponding to the dorsal half of atlas levels 35 and 36. Numbers in lower left correspond to atlas levels (Swanson, 2004). This figure is also available as a separate vector graphics file (Figure S4).

The pattern of LHAjvv and LHAjvd first-order connections was similar (Figure 3)—a similarity accentuated by comparison to the connections of other medial- and perifornical tier LHA regions with which they differed markedly (Goto et al., 2005; Hahn and Swanson, 2010, 2012). However, our analyses also revealed several marked differences, apparent as differences in both the topography (qualitative differences) and relative abundance (quantitative differences) of LHAjvd/v connections. The rostral reach of LHAjvd/v first-order connections was similar, with the outputs of both extending rostrally to about the rostral limit of the infralimbic area (ILA, Figures 4D,E), and sources of input extending slightly farther rostral, to rostral levels of the prelimbic area (PL, Figures 4A,B). In contrast, connections of the LHAjvd were found to extend farther caudally (albeit sparsely) than those of the LHAjvv, to at least as far caudal as the nucleus of the solitary tract (NTS, Figure 4CCC); the caudal-most connections of the LHAjvv were in the pontine central gray (PCG, Figures 4SS–UU).

Morphological features of labeled axons included substantial branching and distinct terminal arbors of varying density. In addition, labeled axons typically had numerous varicosities—sites of potential synaptic contact (Wouterlood and Groenewegen, 1985; Thomson et al., 1996). Approximately 95% of anterograde and retrograde labeling was ipsilateral to injection sites. Contralateral PHAL and CTB labeling generally mirrored the pattern of ipsilateral labeling in several regions, but its abundance varied, and in several regions where ipsilateral labeling was abundant it was essentially (or entirely) absent: It was present in the hypothalamus, thalamus, midbrain, and dorsal parts of the hindbrain; it was absent from the hippocampal formation, and essentially absent from the amygdalar region, and the lateral half of the rostral midbrain. The results of other injection sites illustrated in Figure 1 are considered below, in the *Comparative Analysis* Section.

### LHAjvd and LHAjvv output connections

#### LHAjvd/v outputs to the cerebral cortex

A conspicuous difference was apparent with respect to LHAjvd/v outputs to the cerebral cortex, in that these connections, which target several parts of the cerebral cortex, arise almost exclusively from the LHAjvv. They include inputs to several components of the amygdalar region associated with the cerebral cortex, and foremost among these (in the cortical subplate) are inputs to basomedial- (BMA, posterior part, very dense), and posterior (PA) amygdalar nuclei (Figures 4X–FF, 5A). In the cortical plate, olfactory-related amygdalar components receive a light to moderate input from the LHAjvv, namely the piriform-amygdalar transition area (PAA, Figures 4X–BB), and the posterior part of the cortical amygdalar area (COAp, mostly its lateral zone, Figures 4X–CC). Two other parts of the cerebral cortex also receive a light input from the LHAjvv: The hippocampal formation (HPF, primarily the subiculum and field CA1, Figures 4CC–II), and the prefrontal cortex (a very light input to prelimbic- and infralimbic areas, Figures 4D–G). Where present, input from the LHAjvd to the cerebral cortex includes regions targeted by the LHAjvv (such as ILA, and COAp), but it is of comparatively little amount.

**Figure 5:**
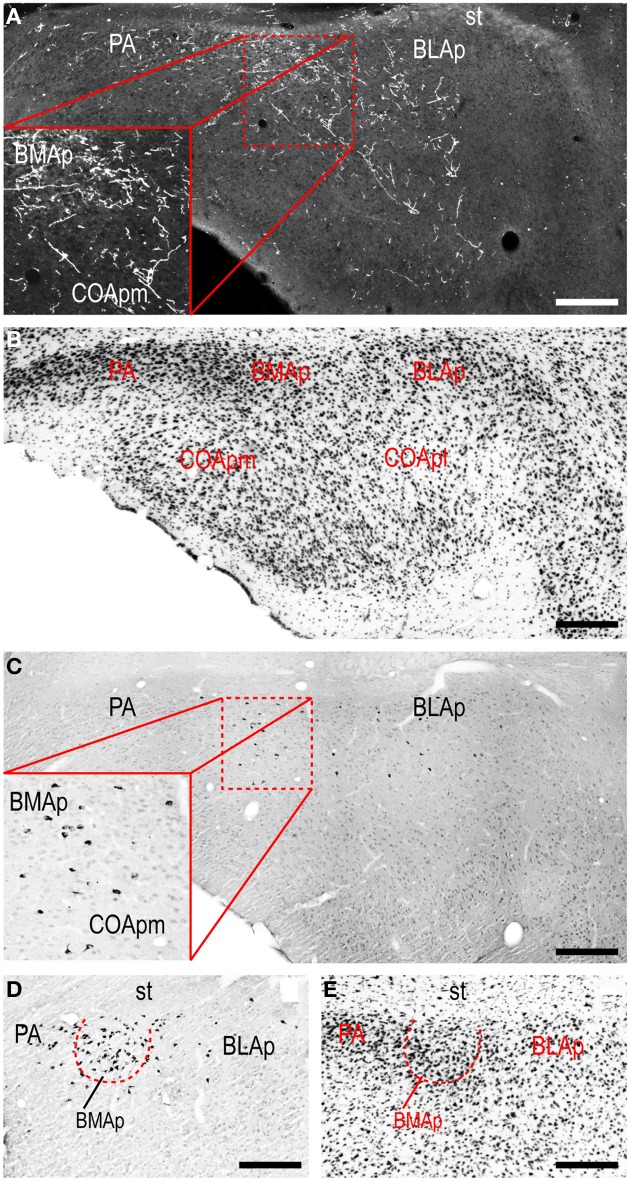
**LHAjvv connections with the cortical parts of the amygdalar region**. Photomicrographs of PHAL/anterograde (**A**, and zoomed inset; darkfield) and CTB/retrograde (**C,D** and zoomed inset in **C**; brightfield) labeling in cortical parts of the amygdalar region. Adjacent sections are shown in **(A,C)**. Adjacent Nissl-stained sections are also shown **(B,E)**. Scale bars = 250 μM **(A–C)**, 200 μM **(D,E)**.

#### LHAjvd/v outputs to the cerebral nuclei

**Figure 6:**
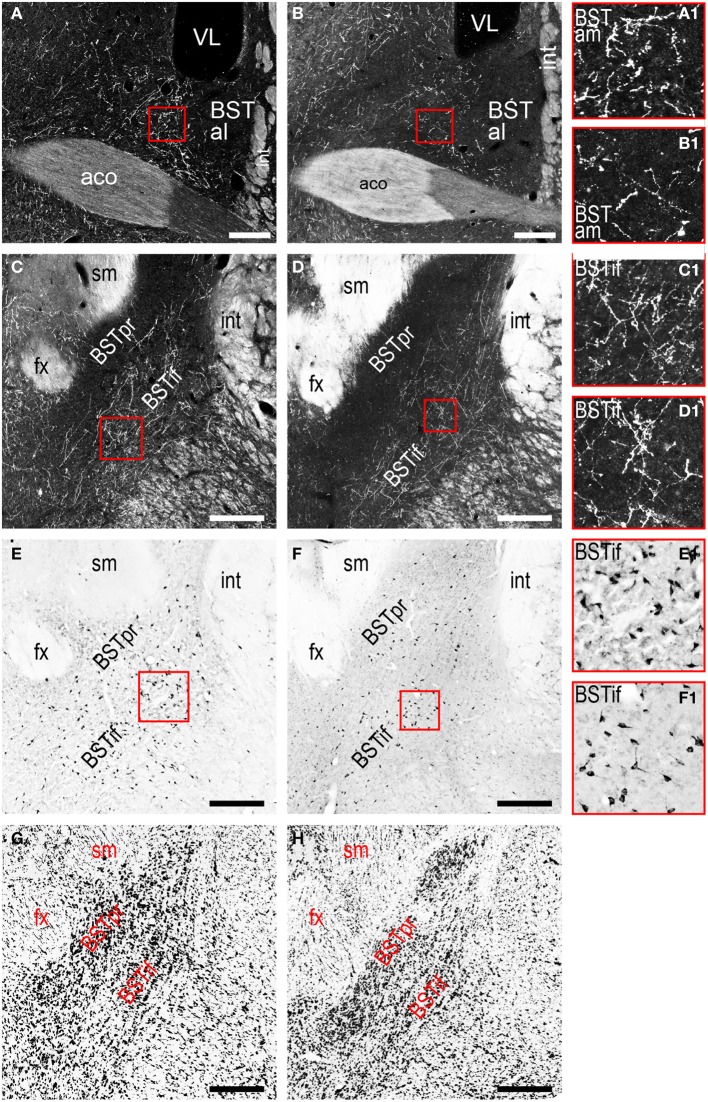
**LHAjv region connections with the bed nucleus of the stria terminalis (BST). (A,B)** Darkfield photomicrographs of PHAL/anterograde labeling in the BST anterior division from the LHAjvd **(A,A1)** and LHAjvv **(B,B1)**. Red boxed areas highlight inputs to the BST anteromedial area. **(C,D)** Darkfield and **(E,F)** brightfield photomicrographs of PHAL/anterograde- **(C,D)** and CTB/retrograde **(E,F)** labeling from the LHAjvd **(C,E,C1,E1)** and LHAjvv **(D,F,D1,F1)** in the BST posterior division. Red boxed areas in **(C–F)** correspond to **(C1–F1)**, and highlight LHAjv region connections with the BST interfascicular nucleus; adjacent sections are shown in **(C,E)** and **(D,F)**; adjacent Nissl-stained sections are also shown **(G,H)**. Scale bars = 250 μM.

**Figure 7:**
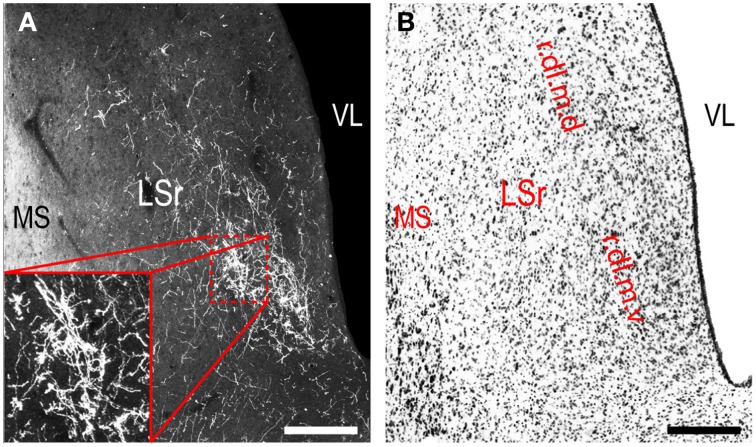
**LHAjvv outputs to the rostral lateral septal nucleus (LSr). (A)** Darkfield photomicrograph of PHAL/anterograde labeling in the LSr from the LHAjvv. Zoomed inset highlights input to the LSr.dl.m.v. **(B)** Adjacent Nissl-stained section. Scale bars = 250 μM.

Beginning with cerebral nuclei of the pallidum, the substantia innominata (SI) receives a substantial input from the LHAjvd/v (Figures 4K–X). This input is present mostly at rostral SI levels, and in the medial half; axons of passage were also observed, intermingled with axon terminals in the SI, especially at caudal levels. More impressive than the SI input is a major input to certain regions of the bed nucleus of the stria terminalis (BST); most striking are inputs to the BSTam (Figures 4L–Q, 6) and BSTif (Figures 4R,S, 7A,B); the LHAjvd also provides a light input to rostral levels of the BSTpr (Figures 4P,Q).

Continuing rostral from the BST into the striatum, a major LHAjvd/v axonal input reaches the lateral septal nucleus (LS), the vast majority of which targets the rostral part (LSr). Within the LSr, each of its three zones (medial, ventrolateral and dorsolateral) receives an input from both LHAjvd and LHAjvv, although the densest input is to the dorsolateral zone (LSr.dl), and especially from the LHAjvv to the ventral domain of its medial region (LSr.dl.m.v) (Figures 4M, 7). The overall extent of input to the LS from the LHAjvd/v is somewhat greater from the LHAjvv.

While most of the LHAjvd/v output to the striatum is to the LS, additional striatal nuclei are also targeted, including (sparingly) the nucleus accumbens (Figures 4G–K), and several striatal components of the amygdalar region, especially the medial part of the central amygdalar nucleus (Figures 4S,T). It is also worth noting that axons were clearly labeled in the stria terminalis, suggesting at least one route whereby axons originating in the LHAjvd/v reach striatal amygdalar nuclei.

#### LHAjvd/v outputs to the cerebrospinal trunk

**Figure 8:**
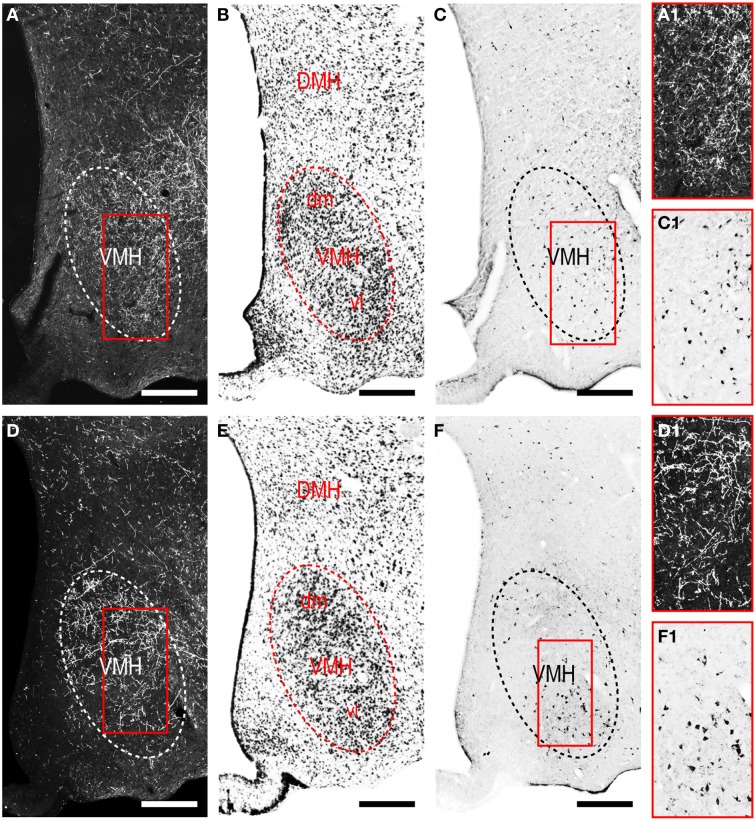
**LHAjv region connections with the ventromedial hypothalamic nucleus (VMH). (A,D)** Darkfield photomicrographs of PHAL/anterograde labeling from the LHAjvd **(A,A1)** and LHAjvv **(D,D1)** in the VMH. Red boxed areas in **(A,D)** correspond to **(A1)** and **(D1)** and highlight LHAjv region inputs to the VMH. **(C,F)** Brightfield photomicrographs of CTB/retrograde labeling from the LHAjvd **(C)** and LHAjvv **(F)** in the VMH. Red boxed areas in **(C,F)** correspond to **(C1,F1)** and highlight LHAjv region inputs to the VMH. Adjacent sections are shown in **(A,C)** and **(D,F)**. Adjacent Nissl-stained sections are also shown **(B,E)**. Scale bars = 250 μM.

The LHAjvd and LHAjvv both send a major output to the ventromedial hypothalamic nucleus (VMH); this forms a massive terminal field in the VMH that extends across the entire nucleus (Figures 4V–Z, 8), including a dense bilateral input to the VMHa (Figure 4V). In contrast, the dorsomedial hypothalamic nucleus (DMH) receives very little input from the LHAjvd, and a relatively sparse input from the LHAjvv (Figures 4X–AA). LHAjvd/v outputs to other LHA regions are comparable (slightly greater from the LHAjvd); they are extensive, but of generally low abundance, with the most prominent being restricted to the LHA anterior group and LHA medial tier regions. Nevertheless, the LHAjp and LHAjd both receive a moderate input from the LHAjvd/v (Figures 4S–Z); also of note is a bidirectional connection between the LHAjvd and LHAjvv (Figures 4T–X).

Tracing rostrally from the LHAjvd/v, the next hypothalamic site to receive substantial input is the anterior hypothalamic nucleus (AHN); this input is mostly restricted to the anterior and central parts of the AHN (Figures 4S–Q). At similar levels, a sparse (mostly LHAjvv) input to the parvicellular division of the PVH is noteworthy (Figures 4S–V). Additional rostral hypothalamic sites receiving a major input from both LHAjvv and LHAjvd include the lateral- (LPO) and (especially) medial (MPO) preoptic areas (Figures 4K–Q). At the caudal end of the hypothalamus, the posterior hypothalamic nucleus (PH) receives a substantial input from the LHAjvv, in comparison to a rather sparser input from the LHAjvd (Figures 4AA,BB) (despite the presence of substantial LHAjvd-originating axons of passage in the PH).

The LHAjvd and LHAjvv both send a moderate to abundant output to thalamic nuclei within the midline group of the dorsal thalamus, specifically to thalamic paraventricular- (PVT; entire length, and somewhat bilateral, Figures 4R–CC) and paratenial (PT; rostral levels, Figures 4R,S) nuclei, and less so to the nucleus reuniens (RE; mostly from the LHAjvv to RE ventral half, and especially anterior part, Figures 4S–Z). In addition, the LHAjvd/v sends an output to the subparafascicular nucleus of the thalamus (SPF), in particular from the LHAjvd to the medial division of the SPF parvicellular part (SPFpm, Figures 4DD,FF).

Caudal to the hypothalamus, the major LHAjvd and LHAjvv output targets are the periaqueductal gray (PAG) and the midbrain reticular nucleus (MRN). In addition, the LHAjvd provides a moderate input to the superior colliculus (SC). Reviewing these connections in more detail, the PAGvl receives an extremely dense and extensive input (mostly restricted to its dorsal half), a more moderate input is received by the PAGd; the PAGm and PAGrm also receive a light to moderate input (Figures 4DD–RR, 9). Furthermore, the precommissural and commissural PAG both receive a moderate input from the LHAjvd, but little input from the LHAjvv (Figures 4DD–II). Input to the MRN (reaching there via the PAG) is directed to its magnocellular part, and is especially dense from the LHAjvd (Figures 4JJ–LL). The moderate input to the SC from the LHAjvd targets mostly its deep gray layer, but also includes lightly SC intermediate layers (Figures 4FF–PP).

**Figure 9:**
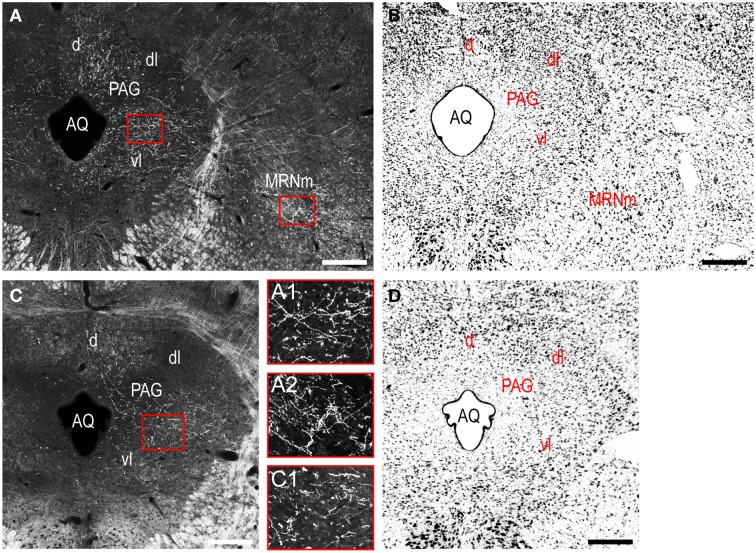
**LHAjv region outputs to the midbrain. (A,C)** Darkfield photomicrographs of PHAL/anterograde labeling in the midbrain from the LHAjvd **(A,A1,A2)** and LHAjvv **(C,C1)**. Red boxed areas in **(A,C)** correspond to **(A1,A2)** and **(C2)**, and delineate inputs to the PAGvl **(A1,C1)** and MRNm **(A2)**. Adjacent Nissl-stained sections are also shown **(B,D)**. Scale bars = 250 μM.

At caudal levels of the PAG, an output from the LHAjvd (moderate) and LHAjvv (light) to the cuneiform nucleus was apparent (Figures 4NN–PP), as was a lighter LHAjvd/v output to the lateral parabrachial nucleus. Caudal to the PAG, input from the LHAjvd/v was relatively light and arose primarily from the LHAjvd; regions receiving input included the pontine central gray (PCG), lateral dorsal tegmental- (LDT) and Barrington's (B) nuclei, locus ceruleus (LC), pontine- (PRN, caudal part) and parvicellular reticular nuclei (PARN), and the nucleus of the solitary tract (NTS, commissural part, Figures 4BBB,CCC).

### LHAjvd and LHAjvv input connections

#### LHAjvd/v inputs from the cerebral cortex

Cerebral cortical retrograde labeling from the LHAjvd/v was abundant; however, it was more abundant for the LHAjvd than for the LHAjvv (in contrast to cerebral cortical input connections from the LHAjvd/v, which arose almost exclusively from the LHAjvv). The sources of this cortical input were within areas and regions of the sensory-motor- and polymodal association cortices. With respect to the former, a moderate amount of retrograde labeling was found in cortical amygdalar- (COA, especially posterior part, lateral zone, Figures 4Z–DD) and infralimbic (ILA, Figures 4D–G) areas, and to a lesser degree in the tenia tecta (TT, dorsal part), postpiriform transition area (TR), and the nucleus of the lateral olfactory tract (NLOT).

Areas of the polymodal association cortex providing input to the LHAjvd/v include the prelimbic- (PL, moderate abundance, Figures 4A–D) and perirhinal (PERI, low abundance) areas, and the hippocampal formation (HPF, high abundance). Within the HPF, a low to moderate amount of retrograde labeling from the LHAjvd (but not the LHAjvv) was found in the entorhinal area, and abundant retrograde labeling (principally from the LHAjvd) was present in the subiculum and field CA1 (Figures 4DD–MM). Cortical subplate retrograde labeling from the LHAjvd/v was copious yet circumscribed, and was localized primarily in basal- and posterior (PA) amygdalar nuclei; more specifically, the basolateral- (BLA, posterior part) and adjacent basomedial [BMA, posterior (mostly) and anterior parts] amygdalar nuclei (Figures 4Z–EE, 5).

#### LHAjvd/v inputs from the cerebral nuclei

**Figure 10:**
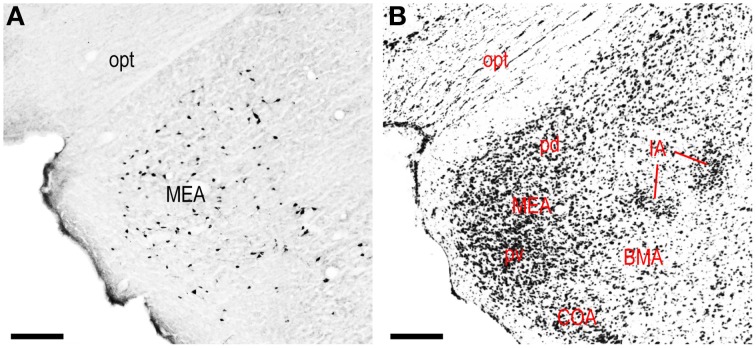
**LHAjvv inputs from the medial amygdalar nucleus (MEA). (A)** Photomicrograph of CTB/retrograde labeling in the MEA from the LHAjvd. An adjacent Nissl-stained section is also shown **(B)**. Scale bars = 200 μM.

Retrograde labeling from the LHAjvd/v was present in several cerebral nuclei. Starting with striatal nuclei, numerous neurons were retrogradely labeled in the rostral part of the lateral septal nucleus (LSr), and in the medial amygdalar nucleus (MEA). Within the LSr, the highest density of retrogradely labeled neurons was present in the LSr ventrolateral zone, followed by the LSr dorsolateral- and medial zones (Figures 4G–O); a little retrograde labeling was also present in the LS caudal part. Retrograde labeling in the MEA was numerous, included each of its parts, and was mostly more abundant from the LHAjvd (Figures 4S–Z, 10); relatively little retrograde labeling from the LHAjvd/v was present in the CEA as well. A low to moderate abundance of retrogradely labeled neurons (moderate from the LHAjvv) was also present in a dorsomedial region of the nucleus accumbens (Figures 4H–K).

Among pallidal nuclei, the most numerous retrograde labeling from the LHAjvd/v was in the following nuclei of the bed nucleus of the stria terminalis: Anteromedial- (BSTam), principal- (BSTpr), and interfascicular (BSTif) nucleus (Figures 4L–S, 6). In fact, the BST as a whole (but especially the BSTam, -pr, and -if) had the highest abundance of retrograde labeling obtained for an individual region in this analysis. In relation to this, it is noteworthy that while analysis of anterograde labeling revealed the BSTam and BSTif both receive a robust input from the LHAjvd/v, the BSTpr receives comparatively little. Retrogradely labeled neurons in other BST nuclei were rarely present.

#### LHAjvd/v inputs from the cerebrospinal trunk

The cerebrospinal trunk was extensively retrogradely labeled from the LHAjvd/v, with the vast majority of retrogradely labeled neurons present in the motor subsystem. Only six regions of the sensory and behavioral state subsystems (three from each) were retrogradely labeled (moderately at most) from the LHAjvd/v; this compares to retrograde labeling at a moderate or higher level in 22 regions of the motor subsystem from the LHAjvd/v. Nevertheless, of the three prominent sensory subsystem input sites, two were retrogradely labeled more numerously from the LHAjvd, and these were in the thalamus: The subparafascicular- (parvicellular part, especially lateral division) and paraventricular thalamic nuclei. The other (and most) notable site of sensory subsystem retrograde labeling from the LHAjvd/v was the parabrachial nucleus, especially its lateral division (Figures 4QQ–TT). Notable sources of behavioral state system input to the LHAjvd/v were the subparventricular zone, LHAd, and the pedunculopontine nucleus (the last mostly retrogradely labeled from LHAjvv).

Motor subsystem retrograde labeling from the LHAjvd/v was very abundant. Most of this input originated in the hypothalamus, and came from hypothalamic medial zone nuclei, the hypothalamic periventricular region, and the reticular formation (including from several other LHA regions). Additional retrograde labeling was present in the central gray (substantial), and in the neuroendocrine motor zone (up to moderate). The pattern of motor subsystem retrograde labeling from the LHAjvd and LHAjvv was essentially similar.

Medial hypothalamic zone nuclei retrogradely labeled abundantly from LHAjvd/v were the AHN (mostly central part, Figures 4R–W) and VMH (all parts, but especially ventrolateral, Figures 4V–Z, 8). Substantial retrograde labeling was also found in the MPN (lateral and medial parts, Figures 4O–S), PMv (Figures 4BB,CC), and the posterior hypothalamic nucleus (PH, Figures 4AA–FF); additional retrograde labeling (up to moderate) was present in all divisions of the PAG (most notably the PAGvl) (Figures 4II–QQ).

Several hypothalamic periventricular region nuclei provide LHAjvd/v input. Numerous retrogradely labeled neurons were found in the medial preoptic area (MPO, Figures 4M–R); a relatively low density (yet cumulatively substantial amount) of retrograde labeling was also present in internuclear areas. In addition, moderate retrograde labeling was present in the median preoptic- (MEPO, Figures 4M–Q), anteroventral periventricular- (AVPV, Figures 4N,O), and dorsomedial hypothalamic (DMH, all parts, but mostly anterior, Figures 4X–AA) nuclei; a less than moderate level of retrograde labeling was found in the anterior hypothalamic area (AHA), and in the anterodorsal/ventral- (ADP/AVP) and periventricular hypothalamic (PV, posterior part) nuclei.

A substantial portion of the reticular formation retrograde labeling from the LHAjvd/v was located in nearby regions of the hypothalamic lateral zone; notably in the LPO (Figures 4K–Q), and in several motor-related LHA regions, including (especially) the LHAav, LHAjp, and LHAjd (Figures 4S–Z). A low to moderate abundance of retrograde labeling was also present in the LHAad, LHAai, LHAsfp, retrochiasmatic area (RCH), and tuberal nucleus (TU). In addition, a moderate abundance of neurons were retrogradely labeled in LHAjvv from the LHAjvd, and vice versa (corroborating the anterograde labeling). Beyond the hypothalamic zone, two retrogradely labeled midbrain nuclei are noteworthy: (1) The midbrain reticular nucleus (MRN, magnocellular part, labeling mostly restricted to a lateral region at rostral levels, Figures 4GG–JJ), which was retrogradely labeled substantially from the LHAjvd (in comparison to substantially fewer MRN neurons retrogradely labeled from the LHAjvv); (2) the cuneiform nucleus, in which a low abundance of retrograde labeling was found from the LHAjvd/v (slightly greater from the LHAjvd) (Figures 4OO–QQ).

Finally, the neuroendocrine motor system was retrogradely labeled to a moderate level of abundance from the LHAjvd/v; the sources of this input were the supraoptic (SO)- hypothalamic paraventricular (PVH)- and arcuate (ARH) nuclei. A substantial cluster of neurons was retrogradely labeled in the SO from the LHAjvv (Figures 4R–T); ARH retrograde labeling from the LHAjvd/v was of low to moderate abundance (Figures 4V–AA); PVH retrograde labeling from the LHAjvd/v was also of low to moderate abundance and was present mostly in the PVH anterior- (PVHap, Figures 4R–T) and medial parvicellular (PVHmpd, Figures 4U–W) parts.

### Comparative analysis

Previous studies have investigated the output and/or input connections of every LHAjv-contiguous region in the rat: LHAjd (Hahn and Swanson, 2012), AHN (Risold et al., 1994), VMH (Canteras et al., 1994; Shimogawa et al., 2014), TU (Canteras et al., 1994; Toth et al., 2010), and LHAsf (Goto et al., 2005). It was therefore possible to compare the present data with the previously published work, to which the reader is also referred. In addition to comparing the principal datasets, we also compared data obtained from experiments with injection sites that included the LHAjvd or LHAjvv, but were less restricted to them (that is, including one or more LHAjv-contiguous region) (Figure 1). Although a detailed comparative analysis of these data was not within the purview of this study, a general review confirmed the existence of distinct differences and similarities between the previously reported connections of the LHAjv-contiguous regions and the LHAjv region; it also confirmed that (without exception) the pattern of labeling obtained from tracer injections that included the LHAjvd/v and one or more LHAjvd/v-contiguous regions, was a combination of the labeling (the indicated connections) resulting from the most restricted LHAjvd/v injections, plus additional connections described previously for the LHAjv-contiguous regions. In addition, the present results are in agreement with a previous preliminary analysis of LHAjv region output connections based on data obtained from a single PHAL experiment (Goto et al., 2005); they are also in general agreement with a recent analysis of inputs to a delineated hypothalamic “aggression” area, which overlaps the LHAjv region (Toth et al., 2010).

A potential source of variability in tract-tracing experiments stems from visual approximation of the effective spread of tracer molecules from an injection site. In the present study, restriction of the principal LHAjvd/v injection sites (experiments LHA #2 and #77) to the LHAjv region is supported by data from a PHAL + CTB study of the connections of the nucleus incertus (NI)—a distinct pontine cell group (Goto et al., 2001). The output connections of the NI include a dense terminal input to the LHAjv-contiguous LHAsf; this input specifically delineates the LHAsfa at a rostrocaudal level corresponding to the center of the injection sites for experiments LHA #2 and #77 (compare our Figure 1 to their Figures 8B, 10I) (Goto et al., 2001). The NI to LHAsfa input arises principally from a relatively cell-diffuse lateral differentiation of the NI, referred to as its diffuse part (NId); a less substantial input arises from a cell-compact medial part (NIc). The previous study (Goto et al., 2001) indicated a virtual absence of input to the LHAjv region from the NId (see their Figures 8B,C), and a very light and diffuse input from the NIc (see their Figures 7D,E). Consistent with this data, and consistent with no appreciable spread of CTB into the LHAsf for experiments LHA #2 and #77, it is salient to note we found no retrograde labeling (from experiments LHA #2 and #77) in the NId, and only a few retrogradely labeled neurons in the NIc (Figures 4TT–VV). Similarly, the amount of anterograde labeling we found in the NI from the LHAjv was miniscule, consistent with a general absence of retrograde labeling in the LHAjvd/v following NI CTB injections, even though the latter did result in retrograde labeling of neurons in LHAjv-adjacent regions (Goto et al., 2001).

## Discussion

A readily grasped description of the hypothalamus divides it into three longitudinal zones, bilateral to the third ventricle. In this schema, the lateral-most zone includes all LHA regions, whereas progressively narrower medial and periventricular zones contain several well defined nuclei (Swanson, 1987). Accumulated experimental evidence supports the existence of segregated networks within the hypothalamic medial zone that are critical for the control of different types of fundamental behavior (Risold et al., 1997; Swanson, 2000). Collectively, the medial hypothalamic zone nuclei within these networks form the rostral segment of a behavior control column, the caudal segment of which is formed by regions centered in the ventromedial midbrain (Swanson, 2000) (see their Figure 10).

In two previous sister papers, using the methods applied in the present study on the LHAjvd and LHAjvv, we described the connections of the LHAjd (Hahn and Swanson, 2012) and LHAjp (Hahn and Swanson, 2010)—the two other regions of the LHA medial tier (Swanson, 2004); the connections of the LHA perifornical tier regions (LHAs and LHAsf) have been investigated similarly: LHAs (Hahn and Swanson, 2010), and LHAsf (outputs only) (Goto et al., 2005). The connections of these LHA regions with, and in relation to, the behavior control column suggested different LHA regions have primary involvement with different behaviors: LHAs with ingestive behavior (Hahn and Swanson, 2010), LHAsfa with defensive or exploratory/foraging behavior (Goto et al., 2005), LHAjp and LHAjd with defensive behavior (Hahn and Swanson, 2010, 2012). The connections of the LHAjvd and LHAjvv follow this general pattern and, as we discuss here, extend it to include reproductive and also aggressive behaviors.

In our previous paper we showed the LHAjd has robust connections with three highly interconnected hypothalamic medial zone nuclei (and subdivisions) (AHN, VMHdm, PMd) involved in defensive (“fight”-or-“flight”) behavior control (Canteras et al., 1997; Risold et al., 1997; Canteras, 2002). By comparison, in the present study we found the LHAjv region has substantially less direct connection with the PMd, but robust connections with the AHN and VMHdm. Moreover, both dorsal and ventral LHAjv zones have considerable connectivity with the PMv, VMHvl, and MPN. The latter three medial zone nuclei/subdivisions, which are also highly interconnected, are prominently sexually dimorphic and central to the control of reproductive behavior (Canteras et al., 1997; Risold et al., 1997; Canteras, 2002).

Retrograde labeling from the LHAjv region (up to moderate for the LHAjvd) in the AVPV continues this theme—the AVPV is another prominently sexually dimorphic nucleus which in female rats has a critical role in reproductive function (Wiegand and Terasawa, 1982). A more direct link to reproductive function is suggested by the moderate retrograde (and light anterograde) labeling within regions adjacent to the AVPV that contain a preponderance of gonadotropin-releasing hormone (GnRH) perikarya (Baker et al., 1975; Witkin et al., 1982; Merchenthaler et al., 1984; Wray and Hoffman, 1986; Silverman et al., 1987). The finding of LHAjvd/v connections with the AVPV and GnRH cell body region in the male rat accords with previous retrograde (Hahn and Coen, 2006) and anterograde (Gu and Simerly, 1997) tracing studies in the female rat. In addition, GnRH-immunopositive axons have been reported in the vicinity of the LHAjvd/v (Merchenthaler et al., 1984).

An association of LHAjvd/v connections with reproductive and defensive behaviors extends beyond the hypothalamus, as evidenced by substantial LHAjvd/v retrograde labeling in the BSTpr and all parts of the MEA. In the rat the MEA and BSTpr are both major recipient sites for olfactory information, notably defensive (or aggressive) and reproductive behavior-relevant pheromonal information relayed from the accessory olfactory bulb (AOB) (Scalia and Winans, 1975; Risold et al., 1997; Simerly, 2002; Mohedano-Moriano et al., 2007). Furthermore, the present data indicate a substantial input to the LHAjvd/v from cerebral cortical components of the amygdalar region (BLAp, BMAp, COApl, COApm, PA) that also receive olfactory input from the main and/or accessory olfactory bulbs (Swanson and Petrovich, 1998; Dong et al., 2001a; Petrovich et al., 2001; Pro-Sistiaga et al., 2007). In addition, feedback modulation of this input is suggested by a striking LHAjvv (but not the LHAjvd) input to the BMAp and PA, and a moderate input to the COA.

Olfactory sensory processing in relation to agonistic and reproductive behaviors is prominent for a macrosmatic animal like the rat (Barnett, 1963); nevertheless, reviewing the amygdalar region components connected to the LHAjvd/v it is pertinent to note also associations with non-olfactory sensory processing, and additional behavioral control. For example, conveyance of auditory and visual information to the BMAp and BLAp (and thence to the LHAjvd/v) may be inferred from a massive input to the BMAp (and substantial input to the BLA) from the lateral amygdalar nucleus (LA) (Pitkanen et al., 1995)^1^, which is a major recipient of thalamic and cerebral cortical inputs involved in visual and auditory processing (McDonald, 1998)^1^. Likewise, there are inputs to the LA, BLA, and BMA from thalamic and cerebral cortical regions centrally involved in the processing of somatosensory, viscerosensory, and gustatory sensory information (McDonald, 1998)^1^. Therefore, polymodal information may be relayed to the LHAjvd/v from several striatal and cortical parts of the amygdalar regions.

In addition to having potential relevance for agonistic and reproductive behaviors, conveyance of polymodal information from the amygdalar region to the LHAjvd/v may also be relevant to ingestive behavior. This possibility is illustrated by a series of recent experiments that employed a behavioral model in which feeding is potentiated by an auditory stimulus previously paired with feeding (Holland et al., 2002). In this model, excitotoxic (NMDA) lesion of the BLA is reported to abolish increased feeding to the conditioned stimulus (CS) (Holland et al., 2002)^2^. In addition, a more recent study using the same behavioral model found a significant increase in CS-associated immediate early gene (IEG) expression in basal amygdalar nuclei retrogradely labeled from the LHA (Petrovich et al., 2005). In the latter study, Fluoro-Gold (FG) injections were targeted to the ventral half of the LHA, but were not restricted to particular LHA regions; nevertheless, partial inclusion of the LHAjv region is suggested by a pattern of amygdalar FG labeling similar to the present data (compare their Figure 4A with our Figures 4AA,BB) (Petrovich et al., 2005). Two related and more recent reports examining IEG expression in this model indicated significant involvement of all basal amygdalar regions (Cole et al., 2013), and also the LHAjv region (plus several other LHA regions) (Cole et al., 2015).

More direct LHAjv region links to ingestive behavior (and metabolism) are indicated by several other LHAjv region connections. One in particular stands out, and is indicated by moderate LHAjvd/v retrograde labeling in the hypothalamic arcuate nucleus (Figures 4V–AA) (Atasoy et al., 2012; Keen-Rhinehart et al., 2013; Sohn et al., 2013). For a consideration of this and other of LHAjv region connections in relation to one particular mode of ingestive behavior, namely drinking, see (Swanson, 2000), and note regions identified in their Figure 11 that are shown here to have connections with the LHAjv region (Table 1 and Figure 3).

**Table 1:** **Connections of the LHAjv region**.

**Extrinsic connections of the LHAjv region**	**Outputs**		**Inputs**	
	**(PHAL)**		**(CTB)**	
	**LHAjvv**	**LHAjvd**	**LHAjvv**	**LHAjvd**
	**(LHA#2)**	**(LHA#77)**	**(LHA#2)**	**(LHA#77)**
**CELL GROUP OR REGION**				
**1. CEREBRUM**				
**1.1. CEREBRAL CORTEX**				
**1.1.1. Cortical Plate**				
Sensory-motor cortex				
Somatomotor areas				
Visceral sensory-motor areas				
infralimbic area (ILA)	++	+	++	+++
Olfactory areas tenia tecta				
Dorsal part (TTd)	−	+	++	++
Postpiriform transition area (TR)	−	−	+	++
Piriform-amygdala transition area (PAA)	++	−	−	−
Nucleus of the lateral olfactory tract (NLOT)	−	−	+	++
Cortical amygdalar area				
Anterior part (COAa)	−	−	++	++
Posterior part				
Lateral zone (COApl)	+++	+	+++	++++
Medial zone (COApm)	++	+	++	++
Polymodal association cortex				
Prelimbic area (PL)	+	−	++	+++
Orbital area				
Ventral part (ORBv)	−	−	+	+
Agranular insular area				
Dorsal part (Ald)	−	−	+	+
Posterior part (Alp)	−	−	+	+
Ectorhinal area (ECT)	−	−	+	+
Perirhinal area (PERI)	+	−	++	+
Hippocampal formation				
Retrohippocampal region				
Entorhinal area				
Lateral part (ENTl)	+	−	−	+
Medial part, dorsal zone (ENTm)	−	−	−	++
Medial part, ventral zone (ENTmv)	−	−	−	+++
Subiculum				
Pyramidal layer (SUB-sp)	++	−	+++	+++++
Hippocampal region				
Ammon's horn				
Field CA1				
Stratum radiatum (CA1sr)	+	−	−	−
Pyramidal layer				
Deep (CA1spd)	+	−	++++	+++++
Superficial (CA1sps)	−	−	+	++
Stratum oriens (CA1so)	++	−	−	−
**1.1.2. Cortical Subplate**				
Claustrum (CLA)	−	−	+	+
Endopiriform nucleus				
Dorsal part (EPd)	+	−	−	−
Lateral amygdalar nucleus (LA)	+	+	+	+
Basolateral amygdalar nucleus				
Anterior part (BLAa)	−	−	+	−
Posterior part (BLAp)	+	+	+++	++++
Basomedial amygdalar nucleus				
Anterior part (BMAa)	−	−	+++	++
Posterior part (BMAp)	+++++	+	+++	++++
Posterior amygdalar nucleus (PA)	++++	+	+++	++++
**1.2. CEREBRAL NUCLEI**				
**1.2.1. Striatum**				
Nucleus accumbens (ACB)	+	+	+++	+
Olfactory tubercle (OT)	−	−	+	−
Lateral septal complex				
Lateral septal nucleus				
Caudal (caudodorsal) part				
Dorsal zone				
Rostral region (LSc.d.r)	+	+	−	+
Lateral region (LSc.d.l)	−	−	+	−
Ventral region (LSc.d.v)	−	−	+	−
Ventral zone				
Medial region				
Dorsal domain (LSc.v.m.d)	−	−	+	−
Ventral domain (LSc.v.m.v)	−	−	+	+
Intermediate region (LSc.v.i)	+	−	+	+
Lateral region				
Dorsal domain (LSc.v.l.d)	+	−	++	++
Ventral domain (LSc.v.l.v)	+	−	+	++
Rostral (rostroventral) part				
Medial zone (LSr.m)	+	−	+	++
Dorsal region (LSr.m.d)	−	−	++	++
Ventral region				
Rostral domain (LSr.m.v.r)	+	++	+++	++
Caudal domain (LSr.m.v.c)	+++	++	++	+
Ventrolateral zone				
Dorsal region				
Medial domain (LSr.vl.d.m)	+++	+	++++	+++
Lateral domain (LSr.vl.d.l)	+	+++	++++	+++
Ventral region (LSr.vl.v)	+	+	++	−
Dorsolateral zone				
Medial region				
Dorsal domain (LSr.dl.m.d)	+++	++	+++	++
Ventral domain (LSr.dl.m.v)	++++	++++	+++	++
Lateral region				
Dorsal domain (LSr.dl.l.d)	+++	+++	+++	+++
Ventral domain (LSr.dl.l.v)	−	+	++	++
Ventral part (LSv)	+	++	++	+++
Septofimbrial nucleus (SF)	+	+	+	−
Anterior amygdalar area (AAA)	+	−	+	+
Central amygdalar nucleus				
Medial part (CEAm)	++	+	+	++
Capsular part (CEAc)	+	−	+	−
Medial amygdalar nucleus				
Anterodorsal part (MEAad)	−	+	++++	++++
Anteroventral part (MEAav)	−	−	++	++++
Posterodorsal part				
Sublayer a (MEApd-a)	−	−	+++	+++
Sublayer b (MEApd-b)	+	−	++	++
Sublayer c (MEApd-c)	−	+	+	++
Posteroventral part (MEApv)	−	−	+++	++++
Intercalated amygdalar nuclei (IA)	+	−	−	+
**1.2.2. Pallidum**				
Substantia innominata (SI)	++++	++++	+++	+++
Medial septal complex				
Medial septal nucleus (MS)	+++	+	++	++
Diagonal band nucleus (NDB)	+	+	+	+++
Bed nuclei of the stria terminalis				
Anterior division				
Anterolateral area (BSTal)	+	+	+	+
Anteromedial area (BSTam)	++++	+++++	++++	+++++
Rhomboid nucleus (BSTrh)	+	−	−	+
Dorsomedial nucleus (BSTdm)	+	+	+	−
Fusiform nucleus (BSTfu)	−	−	+	
Ventral nucleus (BSTv)	++	+	+	++
Magnocellular nucleus (BSTmg)	−	−	+	+
Posterior division				
Principal nucleus (BSTpr)	+	+++	++++	+++++
Interfascicular nucleus (BSTif)	++++	+++++	+++++	+++++
Transverse nucleus (BSTtr)	+	+	+	++
****2. CEREBELLUM****	−	−	−	−
****3. CEREBROSPINAL TRUNK****				
**3.1. SENSORY SYSTEM**				
**3.1.1. Thalamus**				
Sensory-motor cortex related				
Ventral group of the dorsal thalamus				
Subparafascicular nucleus thalamus				
Parvicellular part				
Medial division (SPFpm)	+	+++	−	+
Lateral division (SPFpl)	+	+	+	++
Polymodal association cortex related				
Lateral group of the dorsal thalamus				
Medial group of the dorsal thalamus				
Mediodorsal nucleus thalamus				
Medial part (MDm)	+	+	−	−
Intermediodorsal nucleus thalamus (IMD)	−	+	−	+
Midline group of the dorsal thalamus				
Paraventricular nucleus thalamus (PVT)	+++	++++	+	++
Paratenial nucleus (PT)	+++	+++	−	+
Nucleus reuniens				
Rostral division				
Anterior part (REa)	+++	+	−	−
Ventral part (REv)	+	−	−	−
Lateral part (REl)	++	−	−	−
Median part (REm)	−	−	−	+
Caudal division				
Caudal part (REcp)	+	+	−	−
Intralaminar group of the dorsal thalamus				
Central medial nucleus thalamus (CM)	−	+	−	−
**3.1.2. Visual**	−	−	−	−
**3.1.3. Somatosensory**	−	−	−	−
**3.1.4. Auditory**	−	−	−	−
Nucleus of the lateral lemniscus				
Dorsal part (NLLd)	−	+	−	+
Ventral part (NLLv)	−	−	+	+
**3.1.5. Gustatory**	−	−	−	−
**3.1.6. Visceral**	−	−	−	−
Nucleus of the solitary tract				
Commissural part (NTSco)	−	+	−	−
Medial part, caudal zone (NTSm)	−	−	−	+
Parabrachial nucleus				
Lateral division				
Central lateral part (PBlc)	+	−	+	+
Dorsal lateral part (PBld)	−	−	+	+
External lateral part (PBle)	−	−	−	+
Superior lateral part (PBls)	−	−	++	+
Ventral part (PBlv)	−	−	+	+
Kölliker-Fuse subnucleus	−	−	−	+
Medial division				
Medial medial part (PBmm)	−	+	−	−
**3.1.7. Humerosensory**				
Subfornical organ (SFO)	−	−	−	+
**3.2. BEHAVIORAL STATE SYSTEM**				
Suprachiasmatic nucleus (SCH)	−	−	−	+
Subparaventricular zone (SBPV)	++	+	++	+++
Hypothalamic lateral zone, state related				
Lateral hypothalamic area, Dorsal region (LHAd)	−	+	++	++
Tuberomammillary nucleus, ventral part (TMv)	−	−	−	+
Supramammillary nucleus				
Medial part (SUMm)	+	−	−	+
Lateral part (SUMl)	+	−	+	+
Pedunculopontine nucleus (PPN)	−	+	++	+
Pontine reticular nucleus, rostral part (PRNr)	−	+	−	
Raphé nuclei				
Superior central nucleus raphé, medial part (CSm)	−	−	−	+
Dorsal nucleus raphé (DR)	+	+	−	+
Laterodorsal tegmental nucleus (LDT)	−	+	+	+
Locus ceruleus (LC)	−	+	−	−
**3.3. MOTOR SYSTEM**				
**3.3.1. Behavior Control Column**				
Medial preoptic nucleus				
Lateral part (MPNl)	+	+	+++	+++
Medial part (MPNm)	−	−	+++	+++
Central part (MPNc)	−	−	−	+
Anterior hypothalamic nucleus				
Anterior part (AHNa)	+++	+++	++	++
Central part (AHNc)	+++	+++	+++++	++++
Posterior part (AHNp)	−	+	++	+
Paraventricular nucleus hypothal., descending division				
Dorsal parvicellular part (PVHdp)	−	−	−	+
Lateral parvicellular part (PVHlp)	−	−	+	+
Ventromedial hypothalamic nucleus				
Anterior part (VMHa)	++	++	++	+
Dorsomedial part (VMHdm)	++++	+++++	+++	++++
Central part (VMHc)	+++	+++	+++	+++
Ventrolateral part (VMHvl)	+++	++++	+++++	+++++
Ventral premammillary nucleus (PMv)	−	−	++	+++
Dorsal premammillary nucleus (PMd)	++	−	−	+
Medial mammillary nucleus, median part (MMme)	−	−	−	+
Ventral tegmental area (VTA)	−	−	+	+
Midbrain reticular nucleus, retrorubral area (RR)	−	+	−	−
Midbrain reticular nucleus, parvicellular part (MRNp)	−	++	−	−
**3.3.2. Superior Colliculus, motor related**				
Intermediate gray layer				
sublayer b (SCig-b)	−	+	−	−
sublayer c (SCig-c)	−	+	−	+
Deep gray layer (SCdg)	−	+++	−	−
**3.3.3. Postcerebellar and Precerebellar Nuclei**	−	−	−	−
**3.3.4. Vestibulomotor regions**	−	−	−	−
**3.3.5. Central Gray**				
Epithalamus				
Lateral habenula (LH)	+	−	−	+
Posterior hypothalamic nucleus (PH)	++++	++	++++	++++
Periaqueductal gray				
Precommissural nucleus (PRC)	+	+++	−	+
Commissural nucleus (COM)	++	+++	+	+
Rostromedial division (PAGrm)	+++	++	+	++
Medial division (PAGm)	+	++	−	+
Dorsal division (PAGd)	++++	++++	+	++
Dorsolateral division (PAGdl)	+	+	+	+
Ventrolateral division (PAGvl)	+++++	+++++	++	+++
Pontine central gray, general				
Pontine central gray (PCG)	+	++	+	+
Lateral tegmental nucleus (LTN)	−	−	+	−
Barrington's nucleus (B)	−	+	−	−
**3.3.6. Hypothalamic Periventricular Region**				
Median preoptic nucleus (MEPO)	−	+	+++	+++
Anteroventral periventricular nucleus (AVPV)	−	−	++	+++
Preoptic periventricular nucleus (PVpo)	−	−	+	+
Anterodorsal preoptic nucleus (ADP)	−	−	++	++
Anteroventral preoptic nucleus (AVP)	+	+	++	++
Parastrial nucleus (PS)	−	−	+	+
Medial preoptic area (MPO)	++++	+++++	+++++	+++++
Anterior hypothalamic area (AHA)	++	++	+++	++
Dorsomedial hypothalamic nucleus				
Anterior part (DMHa)	++	+	+++	+++
Posterior part (DMHp)	+	−	+	++
ventral part (DMHv)	++	−	++	++
Periventricular hypothal. nuc., posterior part (PVp)	−	−	++	++
Internuclear area, hypothal. periventricular region (I)	+++	+++	++++	++++
**3.3.7 Reticular Formation**				
Hypothalamic lateral zone, motor related				
Lateral preoptic area (LPO)	+++	+++	++++	+++
Lateral hypothalamic area, motor related (LHAmo)				
Juxtaparaventricular region (LHAjp)	+++	+++	+++	+++
Juxtadorsomedial region (LHAjd)	++	+++	+++	+++
Juxtaventromedial region				
Dorsal zone (LHAjvd)	++	SITE	+++	SITE
Ventral zone (LHAjvv)	SITE	++	SITE	++++
anterior region				
Dorsal zone (LHAad)	++	+++	++	+
Intermediate zone (LHAai)	+	+	++	++
Ventral zone (LHAav)	++	++	++++	+++
Retrochiasmatic area (RCH)	++	++	++	++
Tuberal nucleus (TU)				
Subventromedial part (TUsv)	−	−	++	++
Intermediate part (TUi)	+	−	+	++
Suprafornical region (LHAs)	+	+	+	+
Subfornical region				
Anterior zone (LHAsfa)	−	+	+	+
Posterior zone (LHAsfp)	−	−	+	++
Premammillary zone (LHAsfpm)	−	−	+	−
Magnocellular nucleus (LHAm)	−	−	−	+
Parvicellular region (LHApc)	−	−	−	+
Ventral region				
Medial zone (LHAvm)	−	−	+	+
Lateral zone (LHAvl)	−	−	−	−
Posterior region (LHAp)	+	+	+	+
Preparasubthalamic nucleus (PST)	−	−	−	+
Zona incerta, general				
Zona incerta (ZI)	+	++	+	+
Pretectal region				
Anterior pretectal nucleus (APN)	−	+	−	−
Midbrain reticular nuc., magnocellular part, general				
Midbrain reticular nucleus, magnocellular part (MRNm)	+++	+++++	++	++++
Cuneiform nucleus (CUN)	++	+++	+	++
Pontine reticular nucleus, caudal part (PRNc)	−	+	−	−
Gigantocellular reticular nucleus (GRN)	−	++	−	−
Magnocellular reticular nucleus (MARN)	−	+	−	−
Parvicellular reticular nucleus (PARN)	−	+	−	−
**3.3.8. Motoneuron Groups**				
Neuroendocrine motor zone				
Magnocellular				
Supraoptic nucleus, general				
Supraoptic nucleus, proper (SO)	−	−	+++	+
Paraventricular nuc. hypothal., magnocellular division				
Posterior magnocellular part				
Lateral zone (PVHpml)	−	−	+	−
Medial zone (PVHpmm)	−	−	+	−
Parvicellular				
Paraventricular nuc. Hypothal., parvicellular division				
Anterior parvicellular part (PVHap)	++	+	++	++
Medial parvicellular part, dorsal zone (PVHmpd)	++	+	+	++
Periventricular part (PVHpv)	+	−	+	+
Periventricular hypothalamic nucleus, anterior part (PVa)	−	−	−	+
Periventricular hypothal. nuc., intermediate part (PVi)	−	−	+	+
Arcuate hypothalamic nucleus (ARH)	−	+	++	++

*Connections of the LHAjvd and LHAjvv determined from analysis of CTB + PHAL coinjection experiments. Data from two representative experiments are shown from injections that were centered within the LHAjvd (experiment LHA #77) or LHAjvv (experiment LHA #2). The relative magnitude of output connections (PHAL) is represented by the following semi-quantitative grading schema: - = absence of labeling; + = very low; ++ = low; +++ = moderate; ++++ = high; +++++ = very high. The relative magnitude of input connections (CTB) is represented by the following grading schema: - = absence of labeling; + = 2–8; ++ = 9–22; +++ = 23–44; ++++ = 45–79; +++++ ≥ 80. Regions that contained only PHAL-labeled axons with the appearance of axons of passage (that is, presumptive non-synapse forming), or regions that contained only a single CTB-labeled neuron, or single PHAL-labeled axon terminal, are not included in the table (for these details see Figure 4). The brain region hierarchy follows Swanson (2004)*.

Distinct brain regions with demonstrated involvement in the processing of polymodal information relevant to ingestive, agonistic, and reproductive behaviors are therefore linked by their common connection to the LHAjvd/v. This assertion prompts reiteration of a few related conceptual points that serve as a sort of leitmotif to this discussion. Firstly, experimental evidence associating a given brain region with one type of behavior is not evidence of its preclusion from other behaviors; secondly, ultimately this is because all behaviors result from patterned sequential activation of the motor system, which is evidenced by the fact that different behaviors may involve similar motor patterns. For example, the motor pattern necessary for locomotion may be engaged during ingestive, reproductive or agonistic goal-directed behaviors: Approaching a food source (ingestion), a mate (reproduction), or approaching/avoiding a prey/threat (aggression or defense).

Given that behavior generally serves to support survival in a dynamic environment (external and internal), this favors motor patterns that are attuned to environmental context, either through innate or learned mechanisms. In this regard substantial LHAjvd/v connections with the hippocampal formation (HPF; specifically the ventral part of hippocampal field CA1 and the subiculum) are noteworthy. The HPF has a role in episodic memory and spatial navigation (O'Keefe and Nadel, 1978; Squire, 1992; Morris, 2006; Buzsaki and Moser, 2013), with potential relevance to multiple behaviors. For example, and with respect to the current data, this is evidenced by indicated involvement of ventral field CA1 in ingestive (Kanoski et al., 2011; Hsu et al., 2014; Cole et al., 2015) and defensive (Kjelstrup et al., 2002; Pentkowski et al., 2006; Markham et al., 2010; Wang et al., 2013) behaviors; hippocampal involvement in reproductive behaviors is also suggested (Weiland et al., 1997; Woolley et al., 1997; Pawluski and Galea, 2006).

Complexity and diversity of hippocampal function is reflected in its underlying neural connections (intrinsic and extrinsic), which have an intricate topography (Groenewegen et al., 1987; Risold and Swanson, 1996; Witter, 2006; Cenquizca and Swanson, 2007). Especially relevant to the present data are hippocampal output connections to the lateral septal nucleus (LS) (Swanson and Cowan, 1976, 1977; Risold and Swanson, 1997b; Cenquizca and Swanson, 2007). The LS rostral part (LSr) has robust LHAjvd/v connections (present data); furthermore, a continuity of topographic organization exists such that discrete regions of the LS receiving from discrete hippocampal regions connect with discrete regions of the hypothalamus (Risold and Swanson, 1996, 1997b); this is also reflected in regional differences of LS chemoarchitecture (Risold and Swanson, 1997a). The present data extend the continuity of topographical relations to include direct hippocampal connections with the LHAjvd/v—as was also reported in our previously described connections of the LHAjd, LHAjp and LHAs (Hahn and Swanson, 2010, 2012).

Adding another layer of structural organization are recent genetic data indicating that distinct gene expression domains are superimposed on hippocampal connectional topography (Thompson et al., 2008; Dong et al., 2009; Fanselow and Dong, 2010). A model for how these domains relate to existing knowledge of structural and functional hippocampal organization was provided recently (Strange et al., 2014). In general terms the LHAjvd/v connections with the LSr and ventral field CA1/subiculum are consistent with their putative involvement in social behaviors—for example, social defensive behavior (Faturi et al., 2014); however, a deeper understanding will evidently require experimental investigations that take full account of the new genetic data.

Returning to a more focused consideration of LHAjvd/d connections in relation to specific behaviors, of additional note is the moderate bidirectional LHAjvd/v connection with the parvicellular part of the thalamic subparafascicular nucleus (SPFp). This connection was predominantly with the LHAjvd, and primarily an output from the LHAjvd to the SPFp medial division (SPFpm), although it also involved the SPFp lateral division. The SPFpm is indicated to play a role in sexual behavior, as demonstrated (for example) by elevated SPFpm Fos expression in female rats following intromission, and in male rats following ejaculation (Coolen et al., 1996, 1998)—a suggested interpretation is that the SPFp may play a role in post-copulation inhibition of sexual behavior (sexual satiety) (Veening and Coolen, 2014). Other brain regions showing a pronounced increase in Fos expression in rats following ejaculation include the MPN, BSTpr, and MEApd (Veening and Coolen, 1998). Given that these nuclei were substantially retrogradely labeled from the LHAjvd/v, it reinforces potential (and perhaps primary) involvement of the LHAjv region in reproductive behavior control.

In relation to this, it is relevant to review the generally light LHAjvd/v connection with the nucleus accumbens (ACB); this was essentially restricted to the caudal half of the ACB, mostly dorsomedial (ACBdm), and included a low to moderate amount of retrograde labeling from the LHAjvv. A current view of the ACBdm indicates rostral and caudal structure/function differences associated respectively with behavioral expressions of “liking/pleasure” (rostral) and “disliking/displeasure” (caudal) (Richard et al., 2013; Ho and Berridge, 2014). Moreover, a recent study reported a post-ejaculatory change in the electrophysiological profile of medial ACB neurons, consistent with their involvement in behavioral inhibition, although a rostral-caudal distinction was not drawn (Matsumoto et al., 2012). More generally, the need for careful correlation of ACB functional and structural data is emphasized by fine-grained topographic differences highlighted in recent reappraisals of ACB connections (Thompson and Swanson, 2010; Zahm et al., 2013).

Gene expression data and more recent optogenetic studies indicate a role for the LHAjvd/v in the broad control of social behaviors. Notably, androgen and/or estrogen receptors are highly expressed in each of the aforementioned reproductive-behavior related sites that connect with the LHAjvd/v (Simerly et al., 1990; Shughrue et al., 1997). Furthermore, it was shown recently that a member of the LIM/homeobox (Lhx) gene family (Lhx6) is expressed robustly in LHAjvd/v-connected AOB recipient sites, and in nuclei of the hypothalamic medial zone reproductive behavior related network (Choi et al., 2005); whereas other members of the Lhx gene family are expressed in regions of the LHAjvd/v-connected medial hypothalamic zone defensive behavior related network (Choi et al., 2005). More recently, optogenetic manipulation of estrogen receptor 1-expressing neurons in the VMHvl of male mice identified these neurons as an integrative locus for attack and mounting behavior (Lee et al., 2014). Together, these data support a putative role for the LHAjvd/v in the control of defensive/aggressive (agonistic), and especially reproductive behaviors.

With respect to the robust connections of the LHAjvd/v with the VMH, the dendritic morphology of the latter's neurons raises a salient point. In an elegant study, Eugene Millhouse described the morphology of VMH neurons labeled with the rapid Golgi method (Millhouse, 1973). The dendrites of VMH neurons were found to extend well beyond the boundary of the VMH, particularly ventrolateral (through the tuberal nucleus, to reach the pial surface), and also lateral (into the LHAjv region, extending to about the fornix) (Millhouse, 1973). A corollary of this topography is that synaptic input to the LHAjvd/v may be either to LHAjvd/v neurons, to the dendrites of VMH neurons, or to both. Moreover, this consideration applies elsewhere, not least elsewhere in the LHA, as is finely illustrated in other studies by Millhouse (1969, 1973, 1979) that show dendrites of LHA neurons (including those in the LHAjv region) extending beyond the boundaries of their parent regions (as defined by the current LHA parcellation). In addition, the spatial arrangement of VMH dendrites reported by Millhouse (1973) is supported in the current study by the presence of at least a few PHAL-labeled neuronal cell bodies in the VMH and LHAjv region neighbors following LHAjvd/v-targeted injections, even when the injection site was restricted to the LHAjvd/v (Figure 1). These considerations draw attention to a previous PHAL study of the VMH, not only because of the possible inclusion of the neighboring LHA, but also because of several similarities in the outputs of the VMHvl and the LHAjv region (e.g., to the BSTpr, MPO, and parts of the CEA, MRN, and SPFp) (Canteras et al., 1994). Moreover, in addition to the discussed association with reproductive behaviors, it is noteworthy that the VMHvl and LHAjv regions constitute a substantial portion of a hypothalamic region whose stimulation is associated with aggressive behavioral responses (Hrabovszky et al., 2005), giving additional weight to a possible role for the LHAjv region in control of the aggressive component of agonistic behavior.

The foundational relation of neuronal architecture to function is given further emphasis by another LHAjv region hypothalamic connection, as it relates to axon morphology. As noted earlier, axon varicosities can be sites of synaptic terminals of passage (boutons en passant) (Wouterlood and Groenewegen, 1985; Thomson et al., 1996). Apropos of this point, and deserving of extended discussion, is a cluster of retrograde labeling in the supraoptic nucleus (SO) from the LHAjvv (but not the LHAjvd) (Figures 4R–V); incidentally, an injection site restricted mostly to the tuberal nucleus (our experiment LHA #67, Figure 1) also resulted in substantial SO retrograde labeling (not shown)—these results are consistent with a recent CTB retrograde tracing study in the rat (Toth et al., 2010).

Almost (if not) all SO neurons in the rat are neuroendocrine and express either vasopressin (VAS) or oxytocin (OXY) (Swanson, 1986; Markakis and Swanson, 1997). Axon collaterals of SO neurons with numerous varicosities are reported to exist (Mason et al., 1984), but most are oriented dorsal and dorsolateral to the SO and do not extend far from it (rarely reaching more than a few hundred microns beyond the nucleus) (Mason et al., 1984). Nevertheless, longer non-branching axons of SO neurons en route to the posterior pituitary are also reported to possess numerous varicosities, giving them a beaded appearance (Bargmann and Scharrer, 1951; Randle et al., 1986). Beaded VAS-expressing axons coursing along the same pathway (including through the LHAjvv and tuberal nucleus) have also been reported (DeVries et al., 1985). It is of additional note that neurosecretory axons within the posterior pituitary are beaded too (Bargmann and Scharrer, 1951), and form synaptic terminals of passage (Tweedle et al., 1989). Taken together, these data are consistent with magnocellular neuroendocrine axon varicosities as sites of potential synaptic contact, and support the existence of a little-investigated non-neuroendocrine connection of the supraoptic nucleus.

Of related note are two previous retrograde-tracing studies which indicate that a lateral region of the retrochiasmatic area (just dorsal to the optic tract) has bidirectional SO connections (Thellier et al., 1994a,b); in addition, in one of these studies (Thellier et al., 1994a), following separate injections of four different retrograde tracers into the SO, tracer-labeled neurons were not reported in the LHA region corresponding to the LHAjvv, consistent with the present PHAL data (in that we found no evidence of LHAjvv (or LHAjvd) input to the SO). It is not known at this time whether the neurons we found retrogradely labeled in the SO from the LHAjvv expressed VAS or OXY. However, existing knowledge of the relative distribution VAS- and OXY-expressing SO neurons indicates in general terms that dorsomedial and ventrolateral SO neurons appear to mostly express VAS at rostral and caudal levels respectively (vice versa for OXY), with more intermingling at mid levels (Hou-Yu et al., 1986). On this basis, it's possible that both OXY and VAS expressing neurons were retrogradely labeled from the LHAjvv (Figures 4R–V).

Additional perspective is provided by recalling that neuroendocrine neurons projecting to the posterior pituitary are present in (in addition to the PVH and SO) several sites not thought of as classically neuroendocrine, including the BST, MPO, LPO, ZI, and LHA regions lateral to the fornix (Kelly and Swanson, 1980). This highlights the potential for non-neuroendocrine signaling of otherwise neuroendocrine neurons. In the present study this possibility is supported not only by SO retrograde labeling from the LHAjvv, but also by retrograde labeling from the LHAjvd/v in several divisions of the PVH (Figures 4R–W); we also found PVH retrograde labeling in our previous analysis of LHAs, LHAjp, and LHAjd connections (Hahn and Swanson, 2010, 2012). Exemplifying further the potential contribution to behavioral control of dual neuroendocrine/non-neuroendocrine signaling is a recent study reporting retrograde labeling of VAS-expressing neurons in the PVH and SO following injections of the retrograde tracer Fluoro-Gold into dorsal HPF fields CA1-3 (Zhang and Hernandez, 2013).

To round out this section of the discussion, it is fitting to consider the possible functional role of putative SO input to LHAjvv neurons. An interesting possibility is raised by studies indicating a significant increase in the release of OXY (but not VAS) from SO neurons in subordinate male rats encountering dominant aggressive conspecifics (Wotjak et al., 1996; Engelmann et al., 1999). This finding is made more intriguing by OXY microdialysis data obtained in one of the studies, which revealed a significant encounter-associated increase in OXY in the subordinates in a hypothalamic region just medial to the SO and dorsal to the optic tract (Engelmann et al., 1999)—a region adjacent to the LHAjvv, and within the path of OXY axons en route to the posterior pituitary (Armstrong, 1995); moreover, the increase in OXY release in this region was up to ~320% over basal levels (about double the maximum increase measured from within the SO) and, strikingly, was not associated with an increase in plasma levels of OXY (Engelmann et al., 1999). In the same model, a significant increase in VAS but not OXY release in the PVH was measured (also dissociated from neuroendocrine release) (Wotjak et al., 1996). Neurons we found retrogradely labeled in the PVH from the LHAjvv included some within the magnocellular PVH divisions in which OXY and VAS neurons predominate (Figures 4U,V) (Simmons and Swanson, 2009).

Combining these data elucidates a novel potential coordinating link between defensive and reproductive behaviors consisting of VAS/OXY PVH/SO neurons activated in association with defensive behavioral responses, and their downstream connection to the LHAjvv, which is well placed via its connections to influence reproductive behavior. This possibility is enhanced by evidence in the rat of a direct input from the AOB to a region immediately lateral to the SO in which a high density of SO-neuron dendrites are present (Smithson et al., 1992); in the same study no AOB input to PVH was found (Smithson et al., 1992).

In this context and in relation to the earlier discussion of LHAjv region connections with sites activated in response to sexual behavior (notably ejaculation), established links between OXY release and male sexual behavior are noteworthy. Thus, it is suggested that central actions of OXY may include post-ejaculatory inhibition of sexual behavior (Stoneham et al., 1985). Consistent with this suggestion, OXY-expressing SO neurons show elevated post-ejaculatory levels of Fos, correlated to the rapidity of ejaculation (Pattij et al., 2005); also consistent with this suggestion is increased excitation of OXY-expressing SO neurons in male rats by electrical stimulation of the penile nerve (Honda et al., 1999). However, a dichotomous note is struck by a positive association of neuroendocrine OXY release and sexual behavior (Stoneham et al., 1985), and a putative role for OXY-expressing PVH neurons in penile erection (Veronneau-Longueville et al., 1999).

Shifting focus to discussion of other LHAjvd/v connections in relation to their putative involvement in control of fundamental behaviors, several LHAjvd/v-midbrain connections are worth noting. Foremost is a dense and extensive (mostly LHAjvd) input to the PAGvl (predominantly to its dorsal half), and a substantial input to the PAGd. A current structure/function model of the neuronal network underlying defensive behavioral responses to stimuli indicative of a threat (that is, “fear” responses) co-relates specific PAG regions with behavioral responses to particular threat stimuli (Gross and Canteras, 2012; Canteras and Graeff, 2014). In this model, which is based largely on experimental data from male rats, the PAGd and dorsal half of the PAGvl are indicated to play a role in mediating defensive responses to interoceptive and social threat stimuli (for example and respectively, visceral pain and an aggressive conspecific); whereas the PAGvl (but not the PAGd) and PAGdl are indicated (respectively) to mediate defensive responses to non-visceral pain (for example footshock; PAGvl), and existential threats (for example a predator; PAGdl) (Gross and Canteras, 2012; Canteras and Graeff, 2014).

Such a PAG division of function in relation to defensive behavior could be seen from a Darwinian perspective to align with an indicated role for the LHAjvd/v in control of reproductive behavior, as follows: If activation of LHAjvd/v to PAGvl and PAGd connections increases the threshold for activation of defensive behavioral responses to non-life threatening painful, interoceptive, or social stimuli (which require activation of the PAGvl and/or PAGd) concurrently with activation of other LHAjvd/v connections that promote reproductive behavior, conceivably this could increase the probability of reproduction; whereas, when LHAjvd/v connections involved in promoting reproductive behavior are not active, a concurrent reduction in the threshold for activation of the same defensive responses could benefit survival. Furthermore, inhibition of defensive responses to the existential threat of a predator would have less obvious benefit, whether or not an animal was engaging in reproductive behavior, which is consistent with a very sparse LHAjvd/v to PAGdl connection. Similar hypotheses might also be developed in relation to indicated LHAjvd/v involvement in aggressive behavior (Hrabovszky et al., 2005; Toth et al., 2010).

Also in the midbrain, the LHAjv region (mostly LHAjvd) targets extensively the magnocellular part of the midbrain reticular nucleus (MRNm), and the deep gray layer of the superior colliculus (SCdg); fairly extensive LHAjvd connections with the cuneiform nucleus were also apparent, as was a circumscribed LHAjvd/v connection with the nucleus of the lateral lemniscus (NLL). The LHAjvd/v-MRNm connection is very heterogeneous in terms of its distribution and directionality. At rostral MRNm levels, retrograde labeling from the LHAjvd/v was present in a far lateral MRNm region, ventral to the SPFpl, and intermingled with sparsely distributed of PHAL-labeled axons (Figures 4GG–II). At a mid-rostrocaudal MRNm level, a very dense LHAjvd input to a more central region of the MRNm was evident (Figure 4JJ), surrounded by a less dense but more extensive input that extended caudally (Figures 4KK–MM).

In a previous paper, we discussed the MRNm in relation to its light connections with the LHAjd, which are distinct from and less substantial than MRMm-LHAjvd/v connections (Hahn and Swanson, 2012). As a component of the reticular formation within the motor system, the MRN shares the same classification as the entire hypothalamic lateral zone. This close association is reflected in the diversity of indicated MRN structure and function, and also by a general lack of understanding and absence of consensus regarding both (Rye et al., 1987, 1988; Steininger et al., 1992; Inglis and Winn, 1995; Jhou et al., 2009; Kita and Kita, 2011). The present data underscore the need for investigation of subregional MRN differences.

For the LHAjvd connections with the SCdg, CUN and NLL, somewhat more elaborated associations to control of fundamental behaviors can be drawn. The SC has an established role in orienting movements (notably of the eye and head) to various stimuli (Dean et al., 1989); these include visual stimuli conveyed directly to superficial layers of the SC from retinal ganglion cells (Simon and O'Leary, 1992; Hattar et al., 2006; Morin and Studholme, 2014), and indirectly from visual areas of the cerebral cortex (Kasper et al., 1994); deeper SC layers receive and integrate sensory information from multiple sensory modalities, including visual, auditory, and somatosensory (Meredith and Stein, 1986; Stein et al., 2009).

A recent SC retrograde tracing study in rats suggested the existence of a medial and lateral SC division that the authors correlated respectively to behaviors associated with avoidance (such as defensive avoidance of a predator) and approach (such as aggressive approach of a prey, or social approach) (Comoli et al., 2012); these differences were also correlated to somatosensory and visual sensory inputs, such that input from the upper visual field was correlated to avoidance/medial SC, and input from the lower visual field, and somatosensory input from vibrissae, to approach/lateral SC (Comoli et al., 2012). However, the connections of the specific SCdg region that we have identified here as an LHAjvd-recipient site, were not investigated (Comoli et al., 2012). For comparison, a recent study in macaques reported defensive avoidance behaviors evoked in response to microinjection of the GABA_A_ receptor antagonist bicuculline methiodide at medial and lateral intermediate- and deep layer SC sites (DesJardin et al., 2013). Additional cross-species comparative analysis of topographic differences in SC circuitry may help to elucidate further its functions in relation to behavior.

Several studies indicate a role for the cuneiform nucleus in relation to defensive behavioral responses. For example, rats that engage in defensive “freezing” when placed in a context previously paired with a painful footshock show markedly elevated levels of cuneiform nucleus c-Fos expression compared to controls (Carrive et al., 1997). Similarly, a significant increase in cuneiform nucleus c-Fos expression is seen in rats exhibiting defensive behavior in response to predator-associated odor exposure (Dielenberg et al., 2001). With respect to the earlier discussion concerning the PAG, it is relevant to note a major cuneiform nucleus to PAG connection, with the rostral part targeting primarily the PAGdl, and the caudal part primarily the PAGd and dorsal half PAGvl (Netzer et al., 2011)—our data indicate the LHAjv region (primarily LHAjvd) sends an output to all levels of cuneiform nucleus.

The cuneiform nucleus to PAG connection is also indicated to play an essential role in the physiological changes associated with acute defensive responses, which include increased heart rate and blood pressure, related to inhibition of the baroreceptor reflex. For example, rostral cuneiform nucleus microinjection of the GABA_A_ receptor antagonist bicuculline methiodide results in elevated blood pressure and heart rate, baroreceptor reflex inhibition, and elevated c-Fos in the PAGdl (Netzer et al., 2011); whereas, blockade of GABA_A_ receptors in cuneiform nucleus combined with activation of GABA_A_ receptors in the PAGdl prevents the baroreceptor reflex inhibition, and the cardiovascular changes (Netzer et al., 2011). Another central component of the circuitry involved in these responses is the DMH. Thus, pharmacological DMH disinhibition with bicuculline generates cardiovascular effects comparable to those associated with similar disinhibition of the cuneiform nucleus (McDowall et al., 2006; Netzer et al., 2011). It is noteworthy that these effects are also produced by bicuculline disinhibition of a region corresponding to the LHAs (McDowall et al., 2006). In addition, and as noted for the cuneiform nucleus, DMH c-Fos expression in rats is significantly elevated following predator-associated odor exposure that also generates defensive behavioral responses (Dielenberg et al., 2001).

Previous investigation of DMH outputs with discrete PHAL injections elucidated a very light connection to the cuneiform nucleus (Thompson et al., 1996); in the same study, the presence of numerous DMH-originating axons were noted within the LHA, including in the LHAjv region, but their input to the LHAjv region was considered to be sparse (Thompson et al., 1996); in contrast, the presence of extensive DMH retrograde labeling from the LHAjv region in the present study is indicative of a rather substantial DMH to LHAjv connection. Nevertheless, a dense DMH to perifornical region connection described previously (Thompson et al., 1996) does concur with our previous finding of substantial DMH retrograde labeling from the LHAs (Hahn and Swanson, 2010).

In the earlier discussion of LHAjv-PAG connections, we mentioned a recent model that ascribes different roles to different PAG divisions in relation to different threat stimuli (Gross and Canteras, 2012; Canteras and Graeff, 2014). In the most recent iteration (Canteras and Graeff, 2014), the DMH is included in this model as a mediator of interoceptive threats. This is supported by converging lines of experimental evidence. For example, pronounced DMH c-Fos expression occurs in rats exposed briefly to an environment containing hypercarbic gas (20% CO_2_, 21%O_2_, 59% N_2_) (Johnson et al., 2011). In addition, rats receiving an innocuous infusion of sodium lactate after receiving DMH injection of a GABA synthesis inhibitor (l-allylglycine) show elevated heart rate and blood pressure (Shekhar et al., 1996; Johnson et al., 2008), and also reduced social interaction (Johnson et al., 2008); and reduced exploratory behavior (Shekhar et al., 1996).

Comparisons have been drawn between the effects of DMH disinhibition paired with sodium lactate infusion in rats, and the tendency of sodium lactate infusion to precipitate comparable physiological changes in humans predisposed to panic attacks (Cowley and Arana, 1990). It is noteworthy that pharmacological inhibition of DMH GABAergic signaling in rats without infusion of sodium lactate also reduces social interaction (Johnson et al., 2008) and exploratory behavior (Shekhar, 1993), and increases heart rate and blood pressure (Shekhar, 1993; Keim and Shekhar, 1996; Shekhar et al., 1996); furthermore, DMH injection of bicuculline also increases plasma levels of ACTH, corticosterone and noradrenalin; whereas DMH injection of the GABA_A_ agonist muscimol has opposite physiological and behavioral effects (Shekhar, 1993; Shekhar et al., 1993).

The DMH forms a highly interconnected network with five additional hypothalamic nuclei located at preoptic levels of the hypothalamus [AVPV, median preoptic (MEPO)-, anterodorsal/ventral preoptic (ADP/AVP)-, and parastrial (PS) nuclei] (Thompson and Swanson, 2004) (see their Figures 2, 12). It is suggested these six nuclei may constitute a central neural substrate for the generation of visceromotor patterns. Aside from their striking interconnectedness, this is critically supported by their uniquely differentiated output connections to visceromotor-related cell groups: These include neuroendocrine neurons within the hypothalamic neuroendocrine motor zone, and preautonomic PVH neurons (Thompson and Swanson, 2004) (see their Figure 11). Collectively, inputs to these “visceromotor” nuclei are prominent from the rostral hypothalamic behavior control column, and from extrahypothalamic regions notable for their involvement in the processing of interoceptive (viscerosensory and humerosensory) information (Thompson and Swanson, 2004) (see their Figure 15). Interestingly, all six nuclei also provide an input to the LHAjvd/v (most notably the DMH, AVPV and MEPO) but only one receives appreciable LHAjvd/v input: The DMH (Table 1). An additional and more direct influence of interoceptive information on the LHAjv region is evidenced by sparse retrograde labeling from the LHAjvd in the NTS (viscerosensory, Figures 4AAA,BBB) and the SFO (humerosensory, Figure 4R), and moderate retrograde labeling from the LHAjvd/v in the lateral division of the parabrachial nucleus (viscerosensory, Figures 4QQ–TT) (see also Table 1).

These considerations lead to the recognition that the LHAjvd/v appears well placed to integrate polymodal interoceptive information (via its input connections from the DMH and the other “visceromotor” nuclei), and to convey this information (via its output connections) not only to the behavior control column, but also to midbrain regions associated with behavioral initiation (PAG, cuneiform nucleus). Given the prevailing association of LHAjvd connections with reproductive behavior in the discussion so far, and the earlier-developed hypothesis suggesting circumstances in which defensive behavior might be suppressed for the sake of reproductive behavior; the presentation here of a route whereby elevated viscerosensory threats (homeostatic challenges) could inhibit the LHAjvd/v presents an interesting opposite possibility (which might apply also in regard to the earlier discussed LHAjv inputs associated with ingestive behavior). Furthermore, it was noted earlier that LHAs and DMH disinhibition with bicuculline appears to generate similar cardiovascular and behavioral effects (McDowall et al., 2006; Netzer et al., 2011). This association was recently noted and incorporated into a model for detection and response to interoceptive and social threat stimuli (Canteras and Graeff, 2014) (see their Figure 1); possible LHAjd involvement (which receives DMH input) (Thompson and Swanson, 1998; Hahn and Swanson, 2012) was also suggested. The present data argue for inclusion of the LHAjv region in this model.

The final LHAjvd/v-midbrain connection to be discussed here is the aforementioned connection with the NLL; this is mostly with the LHAjvd and includes an output to the NLL dorsal part (NLLd, Figures 4NN–PP) and an input from the NLL ventral part (NLLv), mostly from a cluster of neurons located dorsally in the NLLv (Figures 4MM–QQ). In light of the discussion so far, this connection, despite its relative lightness, is especially interesting. The NLL is central to auditory processing, receiving major input from the cochlear nucleus and sending a major output to the inferior colliculus (Gonzalez-Hernandez et al., 1996; Cant and Benson, 2003; Pollak et al., 2003). A role for the NLL in reflex acoustic startle is indicated, but the precise NLL region contributing to this reflex is a matter of debate (Davis et al., 1982; Lee et al., 1996). Furthermore, a link to social behavior is indicated by the presence of androgen receptors in the rat NLLv (Simerly et al., 1990). It therefore seems relevant to consider further investigation into the role of the NLL in relation to auditory processing associated with social behaviors, and in relation to the specific topography and neurochemistry of NLL connections.

**Figure 11:**
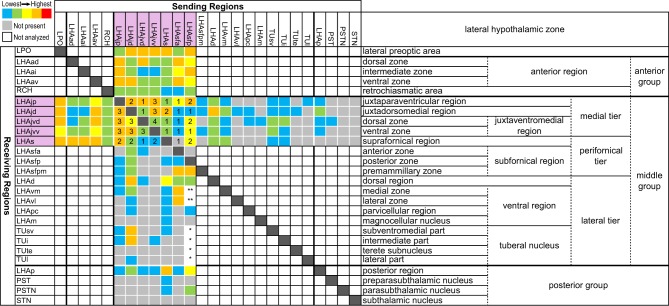
**Matrix of lateral hypothalamic zone connections for all regions of the LHA medial and perifornical tiers in the rat**. Data were obtained from the present and previous studies (Goto et al., 2005; Hahn and Swanson, 2010, 2012). The analysis is restricted to PHAL & CTB data that were mapped directly to the current LHA parcellation. The relative magnitude of output connections for the LHAjp, −jd, −jvd, −jvv, −s, −sfa, and −sfp (PHAL data) and output connections for other parts of the lateral hypothalamic zone (CTB data) are represented by colored squares, according to the five-grade color scale shown, from lowest (blue) to highest (red) (following the scale used in Table 1). Numbered squares indicate the magnitude of retrograde (CTB) labeling (1 = lowest to 5 = highest) for the same direction of connection identified anterogradely with PHAL. Gray squares indicate that analysis was performed but no connection was found. White squares indicate the analysis has not been done. The symbols “^*^” or “^**^” indicate input (PHAL) was weak (^*^) or moderate (^**^), but the subregional distribution was not described.

**Figure 12:**
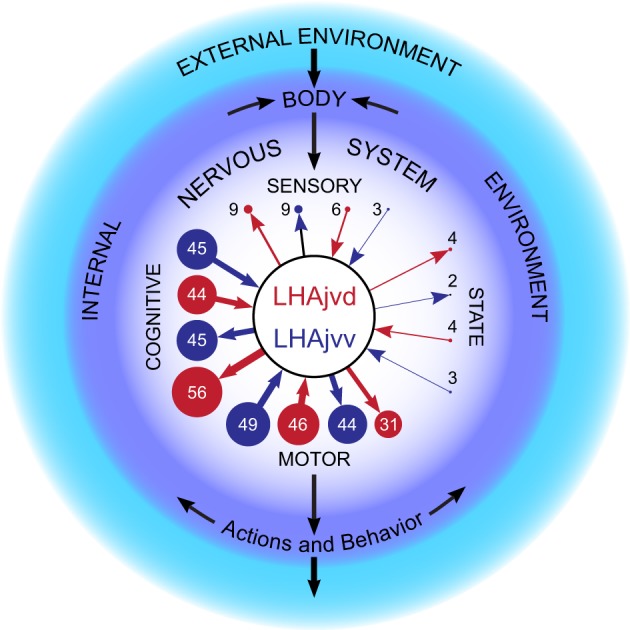
**LHAjv region subsystem connections**. The relative magnitude of LHAjvd (red) and LHAjvv (blue) connections with the sensory, state, cognitive and motor subsystems of the nervous system, presented within in the context of a basic schematic for nervous system functional organization (adapted from Swanson, 2000). Numbers are the percentage for each subsystem (within the central nervous system) of total inputs and outputs (in terms of summated magnitude) (Table 11). The percentages are also represented by the diameter of the discs, and more approximately by arrow line thickness. Note the preponderance of connections with the cognitive and motor systems, and also the bidirectional nature of connections with all subsystems. This figure is also available as a separate vector graphics file (Figure S12).

The extrinsic connections of the LHAjv region are remarkably numerous. In terms of the number of LHAjv-connected gray matter regions, the only comparable examples of this level of connectivity are for the LHAjp, LHAjd, and LHAs (Table 2). Notwithstanding the discussion so far, this exceptional level of connectivity cautions against prediction of functional roles—a caution reinforced by the existence as well of extensive LHA intrinsic connections (Figure 11). In addition, although we have highlighted in this discussion some of the more evident functional associations of the LHAjv region network, a comprehensive model will require synthesis of the available knowledge on each of the several hundred identified LHAjvd/v connections—a non-trivial task. Toward this goal, a high-level perspective is provided by picturing the ratio of LHAjv region connections within the framework of a foundational four subsystem structure-function network model of the nervous system (Swanson, 2000). Placing the present data into this network model makes it apparent that the vast majority of LHAjvd and LHAjvv connections are with the motor and cognitive subsystems (Figure 12), as we also found for the LHAjp, LHAjd, and LHAs (Hahn and Swanson, 2010, 2012). The findings represented in Figure 12 also highlight another general characteristic of LHAjv region connectivity, namely abundant bidirectionality, which is also reflected at the level of individual gray matter regions (Table 1, Figures 3, 4).

**Table 2:** **Connectivity comparison of selected LHA regions**.

	**Number of connected regions**		
	**Outputs**	**Inputs**	**Total**
LHAjp	123	114	237
LHAjd	154	165	319
LHAs	128	130	258
LHAjvd	104	155	259
LHAjvv	104	137	241
ACBdmt	2	5	7

*The number of regional extrinsic input and output connections of selected LHA regions is shown. The data was obtained in the current study, and from previously published works (Hahn and Swanson, 2010, 2012); it is representative of the connections of the selected LHA regions as determined from the following experiments: LHAjp (Exp. #22), LHAjd (Exp. #34), LHAs (Exp. 11), LHAjvd (Exp. #77), and LHAjvv (Exp. #2). For comparison (and contrast) the number of connected regions of the ACBdm dorsal tip (ACBdmt) is also shown (Thompson and Swanson, 2010)*.

Behavioral expression involves the entire nervous system, but it ultimately depends on input from the motor subsystem, which in turn receives input from sensory, cognitive, and behavioral state subsystems (Swanson, 2000). Given that the vast majority of LHAjvd/v connections are with the cognitive and motor subsystems (Figure 12), it follows that LHAjvd/v neurons may play a direct integrating role for information relating to both. Moreover, the extensive bidirectionality of the connections suggests an immediate feedback mechanism. An additional perspective, which also speaks to underlying organizing principles, may be gained by viewing LHAjvd/v connections in the context of an already alluded to structural model for cerebral hemisphere control of motivated behavior (Swanson, 2000).

In relation to the present discussion, the crux of this model is threefold: Firstly, hypothalamic medial zone nuclei form a control column for fundamental goal-oriented behaviors (ingestive, defensive, and reproductive); secondly, the cerebral hemisphere supplies a tripartite descending input to the control column from the cerebral cortex, striatum, and pallidum; thirdly, control column nuclei output to lower levels of the motor subsystem and also (primarily via the thalamus) to the cerebral cortex (Swanson, 2000). In terms of its connections, the LHAjv region conforms to this general model (Table 1, Figure 3). In addition, suggested or indicated functional differentiations for the LHAjvd/v and other medial- and perifornical tier LHA regions (Goto et al., 2005; Hahn and Swanson, 2010, 2012; Betley et al., 2013; Faturi et al., 2014) are congruent with similar functional differentiations for the hypothalamic medial zone behavior control column nuclei (Canteras et al., 1997; Risold et al., 1997; Swanson, 2000; Canteras, 2002). Collectively, these comparisons place the LHA firmly within an existing structure-function model, and point to an indicated role as a coordinator of the behavior control column, and by extension a control-coordinator of fundamental behaviors.

An integrative “high level” behavior coordinating role for the LHAjv region is suggested further by its major bidirectional connections with the BSTif and BSTam (Figure 3). Thorough analysis of the output connections of both indicate they play a controlling or coordinating role in a wide variety of fundamental processes, including (for the BSTif) the control of social behavior (Dong and Swanson, 2004b), and (for the BSTam) the coordination of motor output (neuroendocrine, autonomic, and somatic) relating to energy homeostasis (Dong and Swanson, 2006a). In fact, the outputs of the BST as a whole are practically as diverse as those of the LHA (Dong et al., 2001b; Dong and Swanson, 2003, 2004a,b, 2006a,b,c). To this list might also be added the DMH that is similarly diverse in terms of its inputs, outputs, and implicated functional roles (see earlier discussion) (Thompson et al., 1996; Thompson and Swanson, 1998, 2003). Future retrograde tracing studies of the BST, using the same reference atlas and approach we have applied to the LHA would enable (in combination with existing and present connectivity data) a comprehensive network comparison of the BST and LHA—two similarly diverse and highly differentiated regions.

In addition to knowledge of connections, functional modeling of neuronal networks clearly also requires knowledge of the underlying neurochemistry—in particular the neurotransmitters and their receptors. While it is clear that GABA is the predominant neurotransmitter for extrinsic output connections of the cerebral nuclei (BST included), and glutamate (GLU) for the cerebral cortex (Swanson, 2000), the picture for the LHA is (perhaps unsurprisingly) more complex (Ziegler et al., 2002; Meister, 2007). The challenging complexity is exemplified by the largely LHA-expressed neuropeptides melanin-concentrating hormone (MCH) and hypocretin/orexin (H/O), which are present in intermingled populations of LHA neurons that extend over several LHA regions, including the LHAjvd/v (Swanson et al., 2005; Hahn, 2010). Similarly, neuronal expression of GABA and glutamate is indicated in intermingled populations of LHA neurons within the LHAjv region (although GABA appears to predominate) (Hrabovszky et al., 2005) (see their Figures 2H,I).

Furthermore, MCH and H/O neurons both appear to coexpress either GABA or GLU, but not uniformly: Most (but not all) MCH neurons are indicated to co-release GABA (Elias et al., 2001; Del Cid-Pellitero and Jones, 2012; Jego et al., 2013), but not GLU (Del Cid-Pellitero and Jones, 2012); whereas H/O neurons are indicated to co-release GLU but not GABA (Henny et al., 2010). In addition, a subpopulation of GABAergic (glutamic acid decarboxylase 65-expressing) LHA neurons appear to coexpress neither MCH nor H/O (Karnani et al., 2013). The picture is further complicated by coexpression of neuropeptides. For instance, most (but not all) MCH neurons appear to coexpress cocaine/amphetamine-regulated transcript (CART) (Broberger, 1999; Elias et al., 2001; Cvetkovic et al., 2004), and most (if not all) coexpress the satiety-related neuropeptide nesfatin-1 (Fort et al., 2008); whereas H/O neurons coexpress neither neuropeptide (Elias et al., 2001; Foo et al., 2008; Fort et al., 2008).

Moreover, the neuropeptide gene expression landscape of the LHA is highly pliable. For example, dehydration in rats (brought about by hypertonic saline) results in the neuronal expression of corticotropin-releasing hormone (CRH) in several LHA regions that do not express CRH in euhydrated rats (Kelly and Watts, 1996, 1998; Kay-Nishiyama and Watts, 1999); these include LHA regions in which MCH and H/O are expressed (Swanson et al., 2005; Hahn, 2010), but there is very little co-expression of CRH with either neuropeptide (Watts and Sanchez-Watts, 2007). However, under the same conditions there is a high level of LHA co-expression of CRH and neurotensin (Watts and Sanchez-Watts, 2007). Moreover, most of the dehydration-associated LHA CRH expression occurs in a restricted dorsomedial subregion of the LHAd, with no appreciable CRH expression in the LHAjv region (Watts and Sanchez-Watts, 2007) (see their Figure 2); nevertheless, at least some neurons in this region of the LHAd send an input to the LHAjv region (Figures 4W,X), and overall the LHAd provides a moderate input to the LHAjvd/v (Table 1; compare also our Figure 11 with Figure 2 in Watts and Sanchez-Watts (2007) for review of other dehydration associated CRH-expressing LHA regions in relation to intra-LHA connections).

## Concluding remarks

For a defined gray matter region, the level of LHAjv region extrinsic connectivity is unprecedented. Nevertheless, the connections of the LHAjvd and LHAjvv evidently have a distinct organization (Figure 3). From a systems perspective, they are dominated by bidirectional connections with the motor and cognitive subsystems (Figure 12). At a finer level, they receive substantial input from the cerebral cortex, and cerebral nuclei, as well as providing to both a lesser input (but more substantial for the LHAjvv). They also both have substantial bidirectional connections with medial hypothalamic behavior control column nuclei, and also receive input from hypothalamic nuclei associated with neuroendocrine and visceromotor control. Furthermore, both the LHAjvd and LHAjvv have substantial connections with midbrain regions associated with motor pattern initiation. In essence, the LHAjv region interfaces multiple neural networks necessary for control of voluntary and innate behavior.

With regard to specific behaviors, involvement of LHAjv region connections in control of reproductive behaviors is strongly suggested, but this appears to be integrated with subsidiary involvement in agonistic (defensive/aggressive) and ingestive behaviors. The extent of this involvement appears to be very broad, encompassing somatic, autonomic, and endocrine components of behavior. Similar considerations were raised in our previous investigations of LHAjp, LHAjd, and LHAs connections (Hahn and Swanson, 2010, 2012). These considerations lead us to hypothesize the LHAjv region may constitute part of an LHA hub for controlling the coordination of fundamental behaviors. Integral to this model, in addition to LHA extrinsic connections, are intra-LHA connections (Figure 11). In relation to this, a relevant comparison is with cerebral cortical hubs identified in a very recent informatics analysis of the cerebral cortical association connectome (Bota et al., 2015).

Clearly there is much that remains to be discovered about the structure, organization, and function of LHA neuron populations, and about how the dynamic interplay of these neuron populations within the nervous system as a whole contributes to coordination and control of the rich diversity of behaviors that enable animals to survive and reproduce within their changing and often challenging environments. Rather than attempting an exhaustive and all-inclusive treatment of the several hundred LHAjv region connections identified in this study, we have endeavored to provide a representative overview. Consequently, we realize there may be certain LHAjvd/v connections described in the Results that are of particular interest to individual readers that we have discussed only in passing, or not at all. Nevertheless, the above discussion encompasses a range of perspectives. We hope it sparks associations we may have missed or not addressed, and by the connections we have drawn, illuminates paths to further fruitful research and understanding of the LHA, the brain, and the nervous system.

### Conflict of interest statement

The authors declare that the research was conducted in the absence of any commercial or financial relationships that could be construed as a potential conflict of interest.

## Footnotes

^1^In the cited article a nomenclature different to that used here is employed, such that approximate correspondence may be drawn between BMA and “accessory basal nucleus (AB)”; BLAa/p and “basal nucleus, magnocellular/parvocellular divisions (Bmg/pc).”

^2^In the cited article it is noted that NMDA lesions also partially included adjacent amygdalar regions.

## Abbreviations

AAA: anterior amygdalar area
ab: angular bundle
ACAd: anterior cingulate area, dorsal part
ACAv: anterior cingulate area, ventral part
ACB: nucleus accumbens
aco: anterior commissure, olfactory limb
act: anterior commissure, temporal limb
AD: anterodorsal nucleus, thalamus
ADP: anterodorsal preoptic nucleus
AHA: anterior hypothalamic area
AHNa: anterior hypothalamic nucleus, anterior part
AHNc: anterior hypothalamic nucleus, central part
AHNd: anterior hypothalamic nucleus, dorsal part
AHNp: anterior hypothalamic nucleus, posterior part
Alp: agranular insular area, posterior part
alv: alveus
Alv: agranular insular area, ventral part
amc: amygdalar capsule
AMd: anteromedial nucleus, thalamus, dorsal part
AMv: anteromedial nucleus, thalamus, ventral part
APN: anterior pretectal nucleus
AQ: cerebral aqueduct
AQc: cerebral aqueduct, collicular recess
ARH: arcuate hypothalamic nucleus
AT: anterior tegmental nucleus
AV: anteroventral nucleus, thalamus
AVP: anteroventral preoptic nucleus
AVPV: anteroventral periventricular nucleus
B: Barrington's nucleus
BA: bed nucleus accessory olfactory tract
BAC: bed nucleus anterior commissure
bic: brachium of the inferior colliculus
BLAa: basolateral amygdalar nucleus, anterior part
BLAp: basolateral amygdalar nucleus, posterior part
BMAa: basomedial amygdalar nucleus, anterior part
BMAp: basomedial amygdalar nucleus, posterior part
BSM: bed nucleus stria medullaris
BSTal: bed nuclei stria terminalis, anterior division, anterolateral area
BSTam: bed nuclei stria terminalis, anterior division, anteromedial area
BSTd: bed nuclei stria terminalis, posterior division, dorsal nucleus
BSTdm: bed nuclei stria terminalis, anterior division, dorsomedial nucleus
BSTfu: bed nuclei stria terminalis, anterior division, fusiform nucleus
BSTif: bed nuclei stria terminalis, posterior division, interfascicular nucleus
BSTju: bed nuclei stria terminalis, anterior division, juxtacapsular nucleus
BSTmg: bed nuclei stria terminalis, anterior division, magnocellular nucleus
BSTov: bed nuclei stria terminalis, anterior division, oval nucleus
BSTpr: bed nuclei stria terminalis, posterior division, principal nucleus
BSTrh: bed nuclei stria terminalis, anterior division, rhomboid nucleus
BSTse: bed nuclei stria terminalis, posterior division, strial extension
BSTtr: bed nuclei stria terminalis, posterior division, transverse nucleus
BSTv: bed nuclei stria terminalis, anterior division, ventral nucleus
CA1: field CA1, Ammon's horn
CA1slm: field CA1, lacunosum-moleculare
CA1so: field CA1, stratum oriens
CA1sr: field CA1, stratum radiatum
CA1spd: field CA1, pyramidal layer, deep
CA1sps: field CA1, pyramidal layer, superficial
CA2so: field CA2, stratum oriens
CA2sp: field CA2, pyramidal layer
CA3so: field CA3, stratum oriens
CA3sp: field CA3, pyramidal layer
cc: corpus callosum
ccg: corpus callosum, genu
ccr: corpus callosum, rostrum
CEAc: central amygdalar nucleus, capsular part
CEAl: central amygdalar nucleus, lateral part
CEAm: central amygdalar nucleus, medial part
cic: inferior colliculus commissure
CL: central lateral nucleus, thalamus
CLA: claustrum
CLI: caudal linear nucleus, raphe
CM: central medial nucleus, thalamus
COAa: cortical amygdalar area, anterior part
COApl: cortical amygdalar area, posterior part, lateral zone
COApm: cortical amygdalar area, posterior part, medial zone
COM: periaqueductal gray, commissural nucleus
CP: caudoputamen
cpd: cerebral peduncle
CSl: superior central nucleus raphé, lateral part
CSm: superior central nucleus raphé, medial part
csc: superior colliculus commissure
CUN: cuneiform nucleus
DMHa: dorsomedial hypothalamic nucleus, anterior part
DMHp: dorsomedial hypothalamic nucleus, posterior part
DMHv: dorsomedial hypothalamic nucleus, ventral part
DMX: Dorsal motor nucleus of the vagus nerve
DR: dorsal nucleus, raphe
dscp: superior cerebellar peduncle decussation
DTN: dorsal tegmental nucleus
ec: external capsule
ECT: ectorhinal area
em: external medullary lamina, thalamus
ENTl: entorhinal area, lateral part
ENTm: entorhinal area, medial part, dorsal zone
ENTmv: entorhinal area, medial part, ventral zone
EPd: endopiriform nucleus, dorsal part
EPv: endopiriform nucleus, ventral part
EW: Edinger-Westphal nucleus
fa: corpus callosum, anterior forceps
FF: fields of Forel
fi: fimbria
fr: fasciculus retroflexus
FS: striatal fundus
fx: columns of the fornix
fxpr: precommissural fornix
GPe: globus pallidus, external segment
GPi: globus pallidus, internal segment
GRN: gigantocellular reticular nucleus
I: internuclear area, hypothalamic periventricular region
IA: amygdalar nuclei (intercalated)
IAD: interanterodorsal nucleus, thalamus
IAM: interanteromedial nucleus, thalamus
ICd: inferior colliculus, dorsal nucleus
Ice: inferior colliculus, external nucleus
IF: interfascicular nucleus, raphe
III: oculomotor nucleus
ILA: infralimbic area
IMD: intermediodorsal nucleus, thalamus
INC: interstitial nucleus of Cajal
int: internal capsule
IPNa: interpeduncular nucleus, apical subnucleus
IPNc: interpeduncular nucleus, central subnucleus
IPNd: interpeduncular nucleus, dorsomedial subnucleus
IPNi: interpeduncular nucleus, intermediate subnucleus, IPNld, interpeduncular nucleus, lateral subnucleus, dorsal part
IPNli: interpeduncular nucleus, lateral subnucleus, intermediate part
IPNlr: interpeduncular nucleus, lateral subnucleus, rostral part
IPNr: interpeduncular nucleus, rostral subnucleus
isl: islands of Calleja (olfactory tubercle)
IVn: trochlear nerve
KF: Kölliker-Fuse subnucleus (of parabrachial nucleus)
LA: lateral amygdalar nucleus
LC: locus ceruleus
LDT: laterodorsal tegmental nucleus
LGvm: lateral geniculate complex, ventral part, medial zone
LH: lateral habenula
LHAad: lateral hypothalamic area, anterior region, dorsal zone
LHAai: lateral hypothalamic area, anterior region, intermediate zone
LHAav: lateral hypothalamic area, anterior region, ventral zone
LHAd: lateral hypothalamic area, dorsal region
LHAjd: lateral hypothalamic area, juxtadorsomedial region
LHAjp: lateral hypothalamic area, juxtaparaventricular region
LHAjvd: lateral hypothalamic area, juxtaventromedial region, dorsal zone
LHAjvv: lateral hypothalamic area, juxtaventromedial region, ventral zone
LHAm: lateral hypothalamic area, magnocellular nucleus
LHAp: lateral hypothalamic area, posterior region
LHApc: lateral hypothalamic area, parvicellular region
LHAs: lateral hypothalamic area, suprafornical region
LHAsfa: lateral hypothalamic area, subfornical region, anterior zone
LHAsfp: lateral hypothalamic area, subfornical region, posterior zone
LHAsfpm: lateral hypothalamic area, subfornical region, premammillary zone
LHAvl: lateral hypothalamic area, ventral region, lateral zone
LHAvm: lateral hypothalamic area, ventral region, medial zone
ll: lateral lemniscus
LPO: lateral preoptic area
LSc: lateral septal nucleus, caudal part
LSc.d: lateral septal nucleus, caudal part, dorsal zone
LSc.d.d: lateral septal nucleus, caudal part, dorsal zone, dorsal region
LSc.d.l: lateral septal nucleus, caudal part, dorsal zone, lateral region
LSc.d.r: lateral septal nucleus, caudal part, dorsal zone, rostral region
LSc.d.v: lateral septal nucleus, caudal part, dorsal zone, ventral region
LSc.v.i: lateral septal nucleus, caudal part, ventral zone, intermediate region
LSc.v.l: lateral septal nucleus, caudal part, ventral zone, lateral region
LSc.v.l.d: lateral septal nucleus, caudal part, ventral zone, lateral region, dorsal domain
LSc.v.l.v: lateral septal nucleus, caudal part, ventral zone, lateral region, ventral domain
LSc.v.m.d: lateral septal nucleus, caudal part, ventral zone, medial region, dorsal domain
LSc.v.m.v: lateral septal nucleus, caudal part, ventral zone, medial region, ventral domain
LSr: lateral septal nucleus, rostral part
LSr.dl.l.d: lateral septal nucleus, rostral part, dorsolateral zone, lateral region, dorsal domain
LSr.dl.l.v: lateral septal nucleus, rostral part, dorsolateral zone, lateral region, ventral domain
LSr.dl.m.d: lateral septal nucleus, rostral part, dorsolateral zone, medial region, dorsal domain
LSr.dl.m.v: lateral septal nucleus, rostral part, dorsolateral zone, medial region, ventral domain
LSr.m: lateral septal nucleus, rostral part, medial zone
LSr.m.d: lateral septal nucleus, rostral part, medial zone, dorsal region
LSr.m.v.c: lateral septal nucleus, rostral part, medial zone, ventral region, caudal domain
LSr.m.v.r: lateral septal nucleus, rostral part, medial zone, ventral region, rostral domain
LSr.vl.d.l: lateral septal nucleus, rostral part, ventrolateral zone, dorsal region, lateral domain
LSr.vl.d.m: lateral septal nucleus, rostral part, ventrolateral zone, dorsal region, medial domain
LSr.vl.v: lateral septal nucleus, rostral part, ventrolateral zone, ventral region
LSv: lateral septal nucleus, ventral part
LTN: lateral tegmental nucleus
MA: magnocellular nucleus
MARN: magnocellular reticular nucleus
mct: medial corticohypothalamic tract
MDc: mediodorsal nucleus, thalamus, central part
MDl: mediodorsal nucleus, thalamus, lateral part
MDm: mediodorsal nucleus, thalamus, medial part
MDRNd: medullary reticular nucleus, dorsal part
MDRNv: medullary reticular nucleus, ventral part
ME: median eminence
MEex: median eminence, external lamina
MEAad: medial amygdalar nucleus, anterodorsal part
MEAav: medial amygdalar nucleus, anteroventral part
MEApd.a-c: medial amygdalar nucleus, posterodorsal part, sublayer a–c
MEApv: medial amygdalar nucleus, posteroventral part
MEPO: median preoptic nucleus
MEV: midbrain trigeminal nucleus
MGd: medial geniculate complex, dorsal part
MGm: medial geniculate complex, medial part
MGv: medial geniculate complex, ventral part
MH: medial habenula
ml: medial lemniscus
mlf: medial longitudinal fascicle
MM: medial mammillary nucleus, body
MMme: medial mammillary nucleus, median part
MPNc: medial preoptic nucleus, central part
MPNl: medial preoptic nucleus, lateral part
MPNm: medial preoptic nucleus, medial part
MPO: medial preoptic area
MPT: medial pretectal area
MRNm: midbrain reticular nucleus, magnocellular part
MRNp: midbrain reticular nucleus, parvicellular part
MS: medial septal nucleus
MT: medial terminal nucleus, accessory optic tract
mtV: midbrain tract of the trigeminal nerve
mtt: mammillothalamic tract
NB: nucleus of the brachium, inferior colliculus
NC: nucleus circularis
ND: nucleus of Darkschewitsch
NDB: diagonal band nucleus
NIc: nucleus incertus, compact part
NId: nucleus incertus, diffuse part
NLOT: nucleus of the lateral olfactory tract
NLLd: nucleus of the lateral lemniscus, dorsal part
NLLv: nucleus of the lateral lemniscus, ventral part
NPC: nucleus of the posterior commissure
NTSco: nucleus of the solitary tract, commissural part
NTSl: nucleus of the solitary tract, lateral part
NTSm: nucleus of the solitary tract, medial part
och: optic chiasm
opt: optic tract
ORBm: orbital area, medial part
ORBv: orbital area, ventral part
OT: olfactory tubercle
PA: posterior amygdalar nucleus
PAA: piriform-amygdala transition area
PAGd: periaqueductal gray, dorsal division
PAGdl: periaqueductal gray, dorsolateral division
PAGm: periaqueductal gray, medial division
PAGrl: periaqueductal gray, rostrolateral division
PAGrm: periaqueductal gray, rostromedial division
PAGvl: periaqueductal gray, ventrolateral division
PAR: parasubiculum
PARN: parvicellular reticular nucleus
PBlc: parabrachial nucleus, central lateral part
PBld: parabrachial nucleus, dorsal lateral part
PBle: parabrachial nucleus, external lateral part
PBls: parabrachial nucleus, superior lateral part
PBlv: parabrachial nucleus, ventral lateral part
PBmm: parabrachial nucleus, medial medial part
PBmv: parabrachial nucleus, ventral medial part
pc: posterior commissure
PCG: pontine central gray
PD: posterodorsal preoptic nucleus
PERI: perirhinal area
PF: parafascicular nucleus
PGRNl: paragigantocellular reticular nucleus, lateral part
PH: posterior hypothalamic nucleus
PIR: piriform area
PL: prelimbic area
pm: principal, ammillary tract
PMd: dorsal premammillary nucleus
PMv: ventral premammillary nucleus
PO: posterior complex thalamus
POL: posterior limiting nucleus, thalamus
PP: peripeduncular nucleus
PPN: pedunculopontine nucleus
PPYd: parapyramidal nucleus, deep part
PR: perireuniens nucleus
PRC: periaqueductal gray, precommissural nucleus
PRE: presubiculum
PRNc: pontine reticular nucleus, caudal part
PRNr: pontine reticular nucleus, rostral part
PS: parastrial nucleus
PSCH: suprachiasmatic preoptic nucleus
PST: preparasubthalamic nucleus
PSTN: parasubthalamic nucleus
PSV: principal sensory nucleus of the trigeminal
PT: paratenial nucleus
PVa: periventricular hypothalamic nucleus, anterior part
PVHam: paraventricular nucleus hypothalamus, magnocellular division, anterior magnocellular part
PVHap: paraventricular nucleus hypothalamus, anterior parvicellular part
PVHdp: paraventricular nucleus hypothalamus, dorsal parvicellular part
PVHf: paraventricular nucleus hypothalamus, forniceal part
PVHlp: paraventricular nucleus hypothalamus, lateral parvicellular part
PVHmpd: paraventricular nucleus hypothalamus, medial parvicellular part, dorsal zone
PVHmpdl: paraventricular nucleus hypothalamus, medial parvicellular part, dorsal zone, lateral wing
PVHpml: paraventricular nucleus hypothalamus, posterior magnocellular part, lateral zone
PVHpmm: paraventricular nucleus hypothalamus, posterior magnocellular part, medial zone
PVHpv: paraventricular nucleus hypothalamus, periventricular part
PVi: periventricular nucleus hypothalamus, intermediate part
PVp: periventricular nucleus hypothalamus, posterior part
PVpo: preoptic periventricular nucleus
PVT: paraventricular nucleus, thalamus
py: pyramidal tract
RCH: retrochiasmatic area
Rea: nucleus reuniens, rostral division, anterior part
REcd: nucleus reuniens, caudal division, dorsal part
REcm: nucleus reuniens, caudal division, median part
REcp: nucleus reuniens, caudal division, posterior part
Red: nucleus reuniens, rostral division, dorsal part
REl: nucleus reuniens, rostral division, lateral part
Rem: nucleus reuniens, rostral division, median part
Rev: nucleus reuniens, rostral division, ventral part
RH: rhomboid nucleus
RL: rostral linear nucleus, raphe
RN: red nucleus
RR: midbrain reticular nucleus, retrorubral area
RT: reticular nucleus, thalamus
rust: rubrospinal tract
SAG: nucleus sagulum
SBPV: subparaventricular zone
SCdg: superior colliculus, deep gray layer
SCdw: superior colliculus, deep white layer
SCH: suprachiasmatic nucleus
SCig.a–c: superior colliculus, intermediate gray layer, sublayer a–c
SCiw: superior colliculus, intermediate white layer
scp: superior cerebellar peduncle
sctv: ventral spinocerebellar tract
SF: septofimbrial nucleus
SFO: subfornical organ
SGN: suprageniculate nucleus, thalamus
SH: septohippocampal nucleus
SI: substantia innominate
SLC: subceruleus nucleus
SLD: sublaterodorsal nucleus
sm: stria medullaris
smd: supramammillary decussation
SMT: submedial nucleus, thalamus
SNr: substantia nigra, reticular part
SNc: substantia nigra, compact part
SO: supraoptic nucleus, proper
SOr: supraoptic nucleus, retrochiasmatic part
SPFm: subparafascicular nucleus thalamus, magnocellular part
SPFpl: subparafascicular nucleus thalamus, parvicellular part, lateral division
SPFpm: subparafascicular nucleus thalamus, parvicellular part, medial division
st: stria terminalis
STN: subthalamic nucleus
SUB: subiculum
SUB-m: subiculum, molecular layer
SUB–sp: subiculum, pyramidal layer
SUMl: supramammillary nucleus, lateral part
SUMm: supramammillary nucleus, medial part
sup: supraoptic commissures
SUT: supratrigeminal nucleus
TMd: tuberomammillary nucleus, dorsal part
TMv: tuberomammillary nucleus, ventral part
tp: thalamic peduncles
TR: postpiriform transition area
TRN: tegmental reticular nucleus
TRS: triangular nucleus septum
tsp: tectospinal pathway
TTd: tenia tecta, dorsal part
TUi: tuberal nucleus, intermediate part
TUl: tuberal nucleus, lateral part
TUsv: tuberal nucleus, subventromedial part
TUte: tuberal nucleus, terete subnucleus
V3h: third ventricle, hypothalamic part
V3m: third ventricle, mammillary recess
V3p: third ventricle, preoptic recess
V3t: third ventricle, thalamic part
V4: fourth ventricle proper
VIIn: facial nerve
VL: lateral ventricle
VLP: ventrolateral preoptic nucleus
vlt: ventrolateral hypothalamic tract
VM: ventral medial nucleus, thalamus
VMHa: ventromedial nucleus hypothalamus, anterior part
VMHc: ventromedial nucleus hypothalamus, central part
VMHdm: ventromedial nucleus hypothalamus, dorsomedial part
VMHvl: ventromedial nucleus hypothalamus, ventrolateral part
VPMpc: ventral posteromedial nucleus thalamus, parvicellular part
VTA: ventral tegmental area
vtd: ventral tegmental decussation
VTN: ventral tegmental nucleus
XII: hypoglossal nucleus
ZI: zona incerta
ZIda: zona incerta, dopaminergic group
zl: zona limitans
